# Epidemiology of Traumatic brain injury in Ethiopia: A systematic review and meta-analysis of prevalence, mechanisms, and outcomes

**DOI:** 10.1371/journal.pone.0322641

**Published:** 2025-05-30

**Authors:** Yohannis Derbew Molla, Hirut Tesfahun Alemu

**Affiliations:** 1 Department of Surgery, College of Medicine and Health Sciences, University of Gondar, Gondar, Ethiopia; 2 College of Medicine and Health Sciences, University of Gondar, Gondar, Ethiopia; Duke University Medical Center: Duke University Hospital, UNITED STATES OF AMERICA

## Abstract

**Introduction:**

Traumatic brain injury (TBI) is a major cause morbidity and mortality globally. In Ethiopia, the prevalence of TBI is high, driven by factors such as road traffic incidents (RTIs), assaults, and falls. However, comprehensive data regarding the epidemiology, causes, severity, and outcomes of TBI in Ethiopia remain limited. This scarcity hinders the formulation of evidence-based interventions and policies that are specifically tailored to this context. Therefore, this systematic review and meta-analysis was conducted to provide pooled estimates on the prevalence, causes, severity, complications, and outcomes associated with TBI.

**Method:**

A comprehensive review of existing literature was carried out by searching through PubMed, Scopus, Web of Science and EMBASE to gather studies published until September 2024. The inclusion criteria were centered on studies that discussed the frequency, causes, risk factors and results of brain injury, in Ethiopia. The quality of the studies included in the review was evaluated using the Joanna Briggs Institute (JBI) critical appraisal checklist. We utilized Stata 17 statistical software for analysis. To address the heterogeneity observed among the studies, random-effects models were employed.

**Results:**

The pooled prevalence of TBI among patients presented with trauma was approximately 30.5%. The average age of the patients was 27.48 ± 2.047 years. The main causes for TBI were assaults 36% (CI: 0.28–0.44) road traffic incidents 35% (CI: 0.28–0.41) and falls 21% (CI: 0.15–0.27). Mild TBI made up 57% while moderate and severe TBI accounted for 25% and 18%, respectively. Complications, such as post-traumatic seizures and infections, were reported in 17% of cases. Additionally, the overall mortality rate was 12%. However, significant variability was observed among studies.

**Conclusion:**

The meta-analysis underscores the impact of TBI, in Ethiopia with high mortality rates and associated challenges. The results underscore the pressing requirement for measures to decrease TBIs linked to accidents and violence while also enhancing emergency services access and strengthening neurosurgeons capabilities, in Ethiopian medical facilities.

## Introduction

Traumatic Brain Injury (TBI) is a significant global public health issue, resulting from external mechanical forces that cause temporary or permanent impairments in cognitive, physical, and psychosocial functions. Globally, TBI is a leading cause of morbidity and mortality, with an estimated 69 million cases annually [1]. The burden of TBI is particularly high in low- and middle-income countries (LMICs), where factors such as road traffic incidents, interpersonal violence, and limited access to healthcare exacerbate the problem [2]. Despite its global impact, comprehensive data on TBI in sub-Saharan Africa, including Ethiopia, remain scarce, hindering the development of evidence-based interventions and policies.

The prevalence of TBI varies widely across regions, influenced by differences in injury mechanisms, healthcare access, and preventive measures. In high-income countries, effective trauma care systems and preventive strategies have led to a lower prevalence of TBI among trauma cases [3]. In contrast, LMICs, particularly in sub-Saharan Africa, report higher prevalence rates, often exceeding 30% of trauma cases [4]. For example, studies in Nigeria and Uganda indicate that TBI accounts for 30–40% of trauma cases, driven largely by road traffic incidents and interpersonal violence [5–8]. In Ethiopia, the prevalence of TBI is similarly high, with road traffic incidents, assaults, and falls identified as the leading causes [9,10]. However, the lack of standardized data collection methods and comprehensive studies limits our understanding of the true burden of TBI in Ethiopia.

The impact of TBI extends beyond individual health outcomes, affecting families, communities, and healthcare systems. Globally, TBI is associated with significant morbidity, mortality, and economic costs. Each year, approximately 200,000 individuals with TBI require hospitalization in the United States alone, while an estimated 1.74 million people experience mild TBI that necessitates medical attention or temporary disability [3]. The financial burden of TBI is substantial, with annual costs projected at $4 billion in the United States, encompassing lost income, acute care expenses, and long-term rehabilitation needs [3]. In LMICs, the impact of TBI is even more pronounced due to limited healthcare resources and infrastructure. For example, untreated intracranial injuries, such as epidural hematomas or skull fractures, often result in death or long-term disabilities [11,12]. In Ethiopia, the high mortality rate associated with TBI—estimated at 12% in this study—underscores the urgent need for improved trauma care and preventive measures.

Ethiopia, the second-most populous country in Africa, faces unique challenges in addressing TBI. Rapid urbanization, inadequate road safety measures, and a high prevalence of interpersonal violence contribute to the rising burden of TBI [9,10]. Additionally, the agrarian lifestyle of rural communities increases the risk of TBI due to falls and animal-related injuries [13]. Despite these challenges, detailed data on the prevalence, causes, and outcomes of TBI in Ethiopia remain limited. Previous studies have reported fragmented findings, often focusing on specific regions or populations, which limits their utility for policymakers and healthcare providers. The absence of standardized methodologies and comprehensive reviews further hinders the ability to compare Ethiopia’s TBI burden with that of other LMICs.

This systematic review and meta-analysis aim to address these gaps by pooling available evidence on the prevalence, causes, complications, and outcomes of TBI in Ethiopia. By analyzing trends and comparing them with similar low-resource settings, this study seeks to provide actionable insights to guide policy and improve patient outcomes. The findings will inform targeted interventions to reduce the burden of TBI in Ethiopia, such as road safety initiatives, violence prevention programs, and improvements in trauma care infrastructure.

## Methodology

### Study design

This research presents a systematic review and meta-analysis aimed at investigating the incidence, risk factors, management, and outcomes related to traumatic brain injuries (TBIs) in Ethiopia. The review adheres to the Preferred Reporting Items for Systematic Reviews and Meta-Analyses (PRISMA) guidelines to maintain transparency, rigor, and reproducibility throughout the research process [14].

### Search strategy

A comprehensive literature search was conducted to identify relevant studies published up to September 2024. We conducted a search of electronic databases, including PubMed, Scopus, Web of Science, and EMBASE, utilizing search terms such as “traumatic brain injury,” “TBI,” “head injury,” “Ethiopia,” and other related keywords. To enhance the search process, we employed Boolean operators and Medical Subject Headings (MeSH) terms. Furthermore, In addition to database searches, the reference lists of included articles and relevant reviews were manually reviewed to identify any additional studies that may not have been captured through the initial search. This step ensured comprehensive coverage of all relevant literature on TBI in Ethiopia, particularly studies published in local or regional journals that might not be indexed in major databases. This approach increased the inclusivity and comprehensiveness of the systematic review (S1 Table).

Outcome variables: prevalence of TBI, etiologies of TBI, severity of the trauma (as defined by GCS), type of TBI (isolated/polytrauma), types of intracranial lesion associated with the TBI, complications (posttraumatic seizure, aspiration pneumonia, wound infection and others) and mortality rate (S2 Table).

### Inclusion and exclusion criteria

#### Inclusion Criteria.

- Research studies specifically addressing traumatic brain injuries in Ethiopia.
- Observational, cohort, and case-control studies that evaluate the incidence, risk factors, treatment outcomes, and complications associated with TBIs.
- Studies that present quantitative data regarding patient outcomes, including mortality rates, complications, or functional recovery after experiencing a TBI.

#### Exclusion criteria.

- Studies that are not focused on Ethiopia.
- Case reports, conference abstracts, reviews, and editorial pieces.
**•** Studies that do not provide adequate data or outcome measures pertinent to the objectives of the systematic review.

### Data extraction and quality assessment

Data extraction was conducted independently by two reviewers (HT or YD). Any discrepancies were resolved through discussion or by involving a third reviewer. The data extracted included study characteristics (authors, publication year, study design, and sample size), participant demographics, causes TBI, injury characteristics, management strategies, and outcomes, which encompassed mortality rates, and complications.

Demographic data were extracted from the included studies, which provided information on patient characteristics such as age, gender, and urban/rural residence. These studies were assessed for reliability using the Joanna Briggs Institute (JBI) critical appraisal checklist (S3 Table). Ethiopian census data from the 2007 National Population and Housing Census and subsequent reports were consulted to contextualize and verify demographic distributions where applicable [15]. Additionally, the regional representation of the included studies was cross-referenced with administrative divisions and census data to ensure consistency.

The quality of the studies included in the review was evaluated using the Joanna Briggs Institute (JBI) critical appraisal checklist, which comprises eight questions specifically designed for observational studies. Each study was assessed for potential selection bias, comparability, and the evaluation of outcomes [16]. The Grading of Recommendations Assessment, Development and Evaluation (GRADE) approach was employed to evaluate the certainty of the evidence base (S4 Table). This framework encompasses five key domains: risk of bias, inconsistency, indirectness, imprecision, and publication bias [17].

### Quality assessment and evidence certainty

The quality of the included studies was assessed using the JBI critical appraisal checklist, which revealed that most studies had a low risk of bias in participant selection, measurement methods, and outcome assessment. However, a few studies showed limitations in controlling confounding factors and incomplete reporting of data.

The certainty of evidence, evaluated using the GRADE framework, was rated as moderate to high for most primary outcomes, such as the prevalence of TBI and its etiologies. However, evidence certainty for complications and surgical intervention rates was rated lower due to heterogeneity and inconsistent reporting. These findings underscore the need for more robust and standardized research methodologies in future studies.

### Statistical analysis

The meta-analysis utilized Stata 17 statistical software [18] for its execution. Meta-analysis was performed using random-effects models to account for variability across studies. All demographic and clinical variables were standardized to facilitate comparison. Verification of pooled estimates was achieved by cross-referencing with national hospital data reported by the Ethiopian Federal Ministry of Health (FMOH) where available. Funnel plots and Egger’s tests were employed to assess publication bias, particularly in prevalence estimates. To address the heterogeneity observed among the studies, random-effects models were employed. The main outcome measures included pooled estimates for the prevalence of TBI, injury characteristics, mortality rates, and complication rates. The assessment of heterogeneity was performed using the I² statistic, where values exceeding 50% suggested a notable degree of heterogeneity. To assess publication bias, funnel plots and Egger’s test were utilized.

### Outcome measures

#### Primary outcomes.

1 Pooled prevalence of TBI among trauma cases.
2 Leading causes of TBI, categorized as assaults, RTIs, falls, and others.
3 Overall mortality rate associated with TBI.

#### Secondary outcomes.

1 GCS classification.
2 Complications (post-traumatic seizures, aspiration pneumonia, wound infections).
3 Surgical intervention rate.
4 Imaging findings (skull fractures, epidural hematoma, brain contusion, intracranial hemorrhage).
5 Regional and demographic variations in TBI prevalence and outcomes.

**Short-Term Mortality Rate**: We defined short-term mortality as the proportion of patients who died due to TBI within the timeframe reported by each individual study (e.g., in-hospital mortality or 30-day mortality). This definition accounts for variations in follow-up periods across studies while providing a standardized measure of mortality.

**Complication:** Any adverse events or conditions reported by the included studies that arose after the initial TBI

**Subgroup Analyses** Subgroup analyses were conducted based on study design, region, gender, and place of residence to assess variations in prevalence, complications, and mortality.

**Surgical Intervention Rate** The surgical intervention rate reflects cases requiring neurosurgical procedures based on clinical indications, such as hematomas or elevated intracranial pressure, rather than serving as a direct measure of TBI severity.

### Ethical clearance

‘Clinical trial number: not applicable.’

## Results

### Study characteristics and design

**Fig 1:**
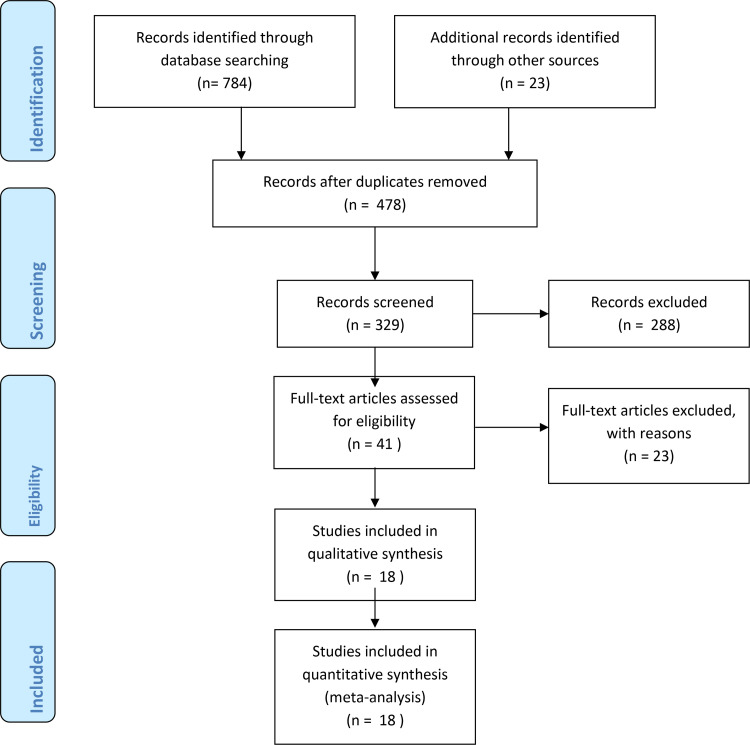
PRISMA flowchart of the study selection process.

Initially, a total of 807 studies were identified. After removing duplicates, 288 studies were excluded based on the established inclusion criteria. The full texts of the remaining 41 studies were thoroughly reviewed, resulting in the inclusion of 18 studies involving 7,854 participants that met the specified inclusion criteria for evidence synthesis (**Fig 1**). Articles were published from 2014 to 2024. The key characteristics of the included studies are presented (Tables 1 and 2). The largest study population consisted of 1,159 individuals, while the smallest included 52. Among the 18 studies reviewed, six were conducted in the Amhara region, four in Addis Ababa, three in Sidama, two in Oromia, two in the Southern Nations, Nationalities, and Peoples’ Region, and one in the Tigray region.

**Table 1:** summary of the confidence intervals.

Parameter		Pooled estimates (%)	Confidence interval (%)
**Sex**	Male	77.9	75.5–80.3
	Female	22.1	19.7–24.5
**Residence**	Urban	46	34 -57
	Rural	54	41- 62
**GCS**	13-15	57	48–65
	9-12	25	19–30
	3-8	18	13–23
**Etiologies**	Interpersonal violence (assault)	36	28 -44
	Road traffic injuries	35	28–41
	Falls	21	15–27
	Others causes (animal kicks, falling objects, etc)	7	5–10

### Patients’ demographics

The average age of the patients was 27.48 ± 2.047 years, with a 95% confidence interval (CI) ranging from 23.18 to 31.78. The demographic analysis of TBI patients across the studies revealed a male predominance, with an overall pooled proportion of 77.9% (± 2.4%), reflecting a male-to-female ratio of approximately 3.5:1. This gender distribution aligns with global trends, where males are more frequently involved in high-risk activities, such as road traffic incidents (RTIs) and interpersonal violence. Studies conducted in urban settings often reported slightly higher male proportions compared to rural areas. Additionally, two studies focused on children, with mean ages of 7.66 ± 3.88 and 9.2 ± 5.4, respectively.

The estimated proportion of patients from rural areas is 0.54 (0.41 to 0.62). In contrast, the proportion of patients from urban areas is 0.46 (0.34 to 0.57). This near-equal distribution highlights the burden of TBI in both settings, albeit with differing etiologies. Rural injuries were predominantly associated with agricultural activities and falls, whereas RTIs and assaults were more prevalent in urban areas. For adults, the proportion stands at 0.73, with a 95% CI of 0.58 to 0.88, and the proportion is 0.27 with a 95% CI of 0.12 to 0.42. It is important to note that significant heterogeneity was observed across the studies. This demographic variability underscores the multifaceted nature of TBI across Ethiopia and highlights the need for tailored interventions addressing both rural and urban contexts (**Table 1**, **S**1 Fig**–**S10 Fig).

### Prevalence of TBI

Five studies have reported on the prevalence of TBI among patients who presented with trauma. The meta-analysis indicates that the prevalence of head trauma in trauma cases is approximately 30.5% (18.5–42.4). However, there is a significant level of heterogeneity, reflected by an I² value of 97%. Among these cases, the prevalence of isolated TBI is estimated at 63% with a 95% confidence interval of 0.53 to 0.73 (S14 Fig–S15 Fig).

### Causes of TBI

A total of 18 studies identifying the causes of TBI were incorporated into the meta-analysis. The findings indicate that assault (interpersonal violence), road traffic incidents (RTIs), and falls are the leading contributors to TBI in Ethiopia, representing 36% (CI: 0.28–0.44), 35% (CI: 0.28–0.41), and 21% (CI: 0.15–0.27) of cases, respectively. Additionally, other causes, including animal kicks, objects falling on the head, firearm-related injuries, and workplace injuries, account for approximately 7% (CI: 0.05–0.10) of TBI cases.. However, there is significant heterogeneity across studies (Tables 2 and 3).

**Table 2:** pooled proportions along with the total sample size (n) for each cause of traumatic brain injury.

Study ID,year	Study Design	Region	Cause: Road Traffic Accidents	Cause: Falls	Cause: Interpersonal Violence	Cause: Occupational Hazards and others	Sample Size
Abebe et al, 2024	Retrospective cohort	Sidama	0.371	0.385	0.2	0.044	1029
Aenderl et al, 2014	prospective	Oromia	0.365	0.154	0.385	0.096	52
Amdeslasie et al, 2017	Retrospective cohort	Tigray	0.249	0.417	0.248	0.049	750
Assele et al, 2021	Retrospective cohort	Sidama	0.611	0.139	0.25	0.01	1159
Ayele et al, 2024	Retrospective cohort	Amhara	0.441	0.128	0.389	0.01	429
Bedry et al, 2020	cross-sectional	Sidama	0.454	0.328	0.126	0.091	317
Biluts et al, 2017	cross-sectional	AA	0.088	0.11	0.758	0.044	91
Demlie et al, 2023	Retrospective cohort	Amhara	0.253	0.138	0.601	0.0074	544
Dibera et al, 2024	prospective	Oromia	0.589	0.109	0.268	0.034	175
Eshete et al, 2018	Retrospective cohort	SERS	0.441	0.198	0.33	0.075	106
Tesfaw et al, 2021	cross-sectional	Amhara	0.248	0.314	0.438	0.03	370
Getabalew et al, 2023	retrospective cross	Amhara	0.24	0.47	0.126	0.163	404
Gezahegn et al, 2019	prospective	SNNE	0.244	0.19	0.29	0.244	90
G/Michael et al, 2023	cross-sectional	Amhara	0.375	0.184	0.242	0.199	483
Hagos et al, 2022	cross-sectional	AA	0.454	0.178	0.283	0.086	304
Landes et al, 2017	prospective	AA	0.41	0.127	0.407	0.049	204
Laeke et al, 2021	cross-sectional	AA	0.158	0.081	0.699	0.061	1087
Walle et al, 2016	cross-sectional	Amhara	0.267	0.124	0.467	0.142	260

### GCS classification

Among patients with TBI, GCS scores were distributed as follows: 57% had scores of 13–15, 25% had scores of 9–12, and 18% had scores of 3–8. It is important to note that these GCS ranges were not used to classify TBI severity, as GCS scores can be influenced by factors such as intoxication, sedation, or systemic conditions. Nonetheless, the heterogeneity is present (S11 Fig–S13 Fig).

Twelve studies examined imaging findings in patients with TBI [19–30]. Among these studies, the prevalence of skull fractures—encompassing linear, depressed, and basilar skull fractures—was reported at 0.29 (95% CI: 0.20–0.38). This was followed by the occurrence of epidural hematoma (EDH) at 0.18 (95% CI: 0.12–0.23), and brain contusion at 0.16 (95% CI: 0.09–0.24). Additionally, the prevalence of intracranial hemorrhage (excluding EDH and contusions) across the included studies was noted to be 0.10 (95% CI: 0.04–0.16). It is important to highlight that there was significant heterogeneity among the studies as only some studies report imaging findings and only some of the patients underwent imaging studies (**Table 3**, S16 Fig**–**S19 Fig).

**Table 3:** Summary of Pooled Estimates and Heterogeneity for Key Variables.

Variable	Pooled Estimate (%)	95% CI	I² (%)	p-value for Heterogeneity	Notes
**Prevalence of TBI**	30.5	18.5–42.4	97	<0.001	High heterogeneity across studies
**Causes of TBI**					
- Assaults	36	28–44	85	<0.001	Regional variations observed
- Road Traffic Incidents (RTIs)	35	28–41	89	<0.001	High heterogeneity
- Falls	21	15–27	78	<0.001	Moderate heterogeneity
- Other Causes (e.g., animal kicks)	7	5–10	82	<0.001	High heterogeneity
**GCS**					
- 13-15	57	48–65	92	<0.001	High heterogeneity
- 9-12	25	19–30	88	<0.001	High heterogeneity
- 3-8	18	13–23	90	<0.001	High heterogeneity
**Imaging Findings**					
- Skull Fractures	29	20–38	84	<0.001	High heterogeneity
- Epidural Hematoma (EDH)	18	12–23	79	<0.001	Moderate heterogeneity
- Brain Contusion	16	9–24	81	<0.001	High heterogeneity
- Intracranial Hemorrhage (non-EDH)	10	4–16	83	<0.001	High heterogeneity
**Complications**					
- Overall Complications	17	10–24	98.79	<0.001	Very high heterogeneity
- Post-traumatic Seizures	12	6–18	95	<0.001	High heterogeneity
- Aspiration Pneumonia	8	4–12	93	<0.001	High heterogeneity
- Wound Infections	6	2–10	91	<0.001	High heterogeneity
**Surgical Intervention Rate**	31	23–40	94	<0.001	High heterogeneity
**Mortality Rate**	12	6–17	95	<0.001	High heterogeneity

**Pooled Estimates**: These are the overall percentages derived from the meta-analysis, **95% CI**: The confidence intervals indicate the precision of the pooled estimates, **I²**: Represents the percentage of total variation across studies due to heterogeneity. Values above 50% indicate significant heterogeneity, **p-value for Heterogeneity**: Indicates whether the observed heterogeneity is statistically significant, **Notes**: Highlights key observations, such as regional variations or very high heterogeneity.

### Complications

Six studies have examined the overall complications associated with TBI, including posttraumatic seizures, aspiration pneumonia, and wound infections [19–21,23,25,26]. The overall prevalence of these complications is reported to be 0.17 (95% CI: 0.1–0.24). Additionally, among patients with TBI, the estimated surgical intervention rate was 31% (95% CI: 23–40%). It is important to note that surgical intervention is not a direct measure of TBI severity but is instead based on specific clinical indications, such as the presence of epidural or subdural hematomas, midline shift, or elevated intracranial pressure. The considerable heterogeneity among studies (I² = 98.79%) may reflect differences in clinical practices, resource availability, and patient populations. It is important to note that there is considerable heterogeneity among the studies, evidenced by an I² value of 98.79%. The meta-analysis examining mortality rates among patients with TBI in Ethiopia, estimates an overall mortality rate of 0.12 (95% CI: 0.06–0.17) (**Table 3**, S20 Fig–S24 Fig, S26 Fig).

### Subgroup analysis

The subgroup analysis revealed substantial heterogeneity in retrospective and cross-sectional studies, with I² values of 96.13% and 97.96%, respectively. This variability may arise from differences in study populations, methodologies, geographical factors, and data collection techniques. In contrast, prospective studies demonstrated no heterogeneity (I² = 0.00%), which may reflect the smaller number of prospective studies included in the analysis, as well as similarities in their populations and methodologies. For example, the prospective studies were conducted in similar urban settings and utilized standardized data collection protocols, which may have contributed to the observed consistency. However, further research is needed to confirm whether these factors fully explain the lack of heterogeneity in prospective studies. The overall heterogeneity remains high (I² = 98.34%), underscoring the need for caution when interpreting pooled results.

The region-based subgroup analysis highlights significant variations in the causes of traumatic brain injury (TBI) across Ethiopia. Road traffic accidents emerge as a major contributor to TBI across all regions, with Sidama and Amhara exhibiting notably higher effect sizes, emphasizing the critical need for targeted road safety interventions in these areas. Falls also play a significant role in TBI occurrences, with Amhara and Sidama regions showing larger effect sizes compared to others, indicating that falls are a more prominent cause of TBI in these regions. Interpersonal violence, while displaying high overall heterogeneity as a cause of TBI, stands out in the Amhara region with a strikingly large effect size, suggesting the need for targeted interventions to address violence-related TBI in this region. Additionally, occupational hazards appear to be a significant contributor to TBI in the Addis Ababa (AA) region, where a markedly higher effect size was observed, underscoring the importance of workplace safety measures to reduce TBI incidence in this area.

The subgroup analyses based on sex and place of residence, while not revealing statistically significant differences between groups for the examined causes of TBI, consistently demonstrate high heterogeneity (I² values) within the subgroups. This observation further emphasizes the complexity of TBI causation in Ethiopia and suggests that additional factors beyond sex and residence likely contribute to the observed variability in the data.

Pooled mortality rates were similar between males (446.7, 95% CI: 276.0–617.4) and females (446.7, 95% CI: 276.0–617.4), suggesting no significant sex-based disparity. However, high heterogeneity (I² = 100%) indicates potential study-level differences in severity and treatment access. Urban patients had a pooled mortality estimate of 406.7 (95% CI: 245.3–568.1), whereas rural patients had 446.7 (95% CI: 276.0–617.4). The slightly higher mortality in rural areas may reflect delays in hospital arrival and limited neurosurgical access. The overall complication rate was 16.7% (95% CI: 9.6–23.8), with some regional variation. The significant regional variation suggests differences in case severity, post-TBI care, or reporting standards. Complication rates varied by study design, with retrospective studies reporting 13.4% (95% CI: 6.1–20.7), cross-sectional studies 17.0% (95% CI: 14.8–19.2), and prospective studies 31.4% (95% CI: 24.5–38.3). The higher rate in prospective studies may indicate better follow-up and documentation.

The operation rate varied significantly by region (Q_b = 131.31, p < 0.0001), with Oromia reporting the highest proportion of surgically managed cases (71.3%, 95% CI: 62.8% – 79.8%**),** while the South East Region (SERS) had the lowest (17.0%, 95% CI: 9.8% – 24.2%) suggesting potential disparities in access to surgical care, healthcare infrastructure, and clinical decision-making practices across different regions. The operation rate was highest in prospective studies (44.3%, 95% CI: 12.3% – 76.2%), followed by retrospective studies (32.7%, 95% CI: 22.0% – 43.3%) and cross-sectional studies (26.8%, 95% CI: 20.5% – 33.2%). The significant heterogeneity (Q_b = 57.58, p < 0.0001) suggests that prospective studies, which often follow patients over time, may have more comprehensive case documentation and higher surgical intervention rates compared to retrospective or cross-sectional designs.

The subgroup analysis did not reveal any significant differences in operation rates between males and females, with both groups having a pooled estimate of 31.4% (95% CI: 22.9% – 39.8%). This suggests that sex is not a determining factor in surgical management within the studied populations. Patients residing in urban areas had a higher operation rate (40.6%, 95% CI: 24.5% – 56.8%) compared to those in rural areas (31.4%, 95% CI: 22.9% – 39.8%). The observed difference may reflect better access to specialized surgical services, higher referral rates, and improved healthcare infrastructure in urban settings. The high degree of heterogeneity (I² = 98.79%) suggests substantial variations between studies, likely due to differences in healthcare access, disease severity, study methodology, and population characteristics.

In summary, the subgroup analyses highlight substantial heterogeneity in study designs and regional causes of TBI in Ethiopia. Variability across retrospective and cross-sectional studies emphasizes the impact of methodological differences, while the consistency in prospective studies underscores their reliability. Regional variations, particularly the high contributions of road traffic accidents, falls, interpersonal violence, and occupational hazards in specific regions, point to the need for region-specific public health interventions. Additionally, the high heterogeneity observed within subgroups based on sex and residence suggests that complex, multifactorial influences underlie TBI causation, warranting further investigation into other contributing factors.

### Heterogeneity

- The pooled prevalence of TBI showed significant heterogeneity (I² = 97%, p < 0.001).
- Heterogeneity was also high for causes of TBI: assaults (I² = 85%, p < 0.001), road traffic incidents (I² = 89%, p < 0.001), and falls (I² = 78%, p < 0.001).
- Similar levels of heterogeneity were observed for severity, complications, and mortality rates (I² > 90%, p < 0.001).

### Publication bias

- Egger’s regression test indicated no significant publication bias for causes of TBI: road traffic accidents (p = 0.5269), falls (p = 0.9818), and occupational hazards (p = 0.3763).
- However, the funnel plot for the prevalence of TBI suggested potential asymmetry, indicating possible publication bias or small-study effects (S25 Fig, S27 Fig).

## Discussion

The estimated prevalence of TBI in trauma cases within our study is 30.5%, which corresponds with the global incidence of TBI. However, it is important to note that this estimate is accompanied by significant heterogeneity (I² = 97%), indicating substantial variability across studies. This variability may be attributed to differences in regional settings, study designs, and data collection methods. While the pooled estimate provides a useful summary of the available data, the high heterogeneity underscores the need for caution when interpreting these findings and generalizing them to the entire Ethiopian population.

The potential publication bias suggested by the funnel plot for TBI prevalence warrants careful consideration, smaller studies with larger effect sizes may disproportionately influence the pooled estimates, leading to an overestimation or underestimation of the true prevalence. While Egger’s test indicated no significant publication bias for the causes of TBI, the possibility of bias in prevalence estimates underscores the importance of including a diverse range of studies in future meta-analyses.

Worldwide, the prevalence of TBI among trauma cases varies considerably, affected by factors such as the mechanisms of trauma, access to healthcare, and the specific risks associated with injuries in different regions. For example, research conducted in high-income nations generally indicates a lower prevalence of TBI among trauma cases, primarily attributable to effective preventive strategies and sophisticated trauma care systems [3]. Conversely, areas with socioeconomic conditions akin to those in Ethiopia, including other regions of sub-Saharan Africa, tend to exhibit similar or even elevated prevalence rates of TBI in trauma incidents, largely influenced by high occurrences of road traffic injuries (RTIs) and insufficient trauma care resources [31].

In African contexts, the prevalence of TBI among cases of trauma shows considerable variation but consistently remains elevated, highlighting the substantial influence of road traffic incidents and injuries related to violence [4,32]. For example, research conducted in Nigeria and Uganda indicates that TBI prevalence rates among trauma cases fall between 30% and 40%, which closely corresponds with our findings [33–36]. The elevated rates of TBI, especially in areas experiencing frequent RTIs, emphasize the pressing need for targeted interventions, including more rigorous enforcement of traffic regulations and enhanced public awareness initiatives regarding road safety.

In our study, the prevalence of isolated TBI was found to be 63%, which aligns with findings from comparable environments in Africa and low- to middle-income countries. In these regions, a notable percentage of TBI cases occur without accompanying polytrauma. Research indicates that this elevated rate of isolated TBI is frequently attributed to prevalent injury mechanisms, such as falls or direct impacts during road traffic incidents (RTIs), which often lead to head injuries in the absence of additional trauma [2,37] Conversely, research conducted in high-income nations indicates that isolated TBI rates are lower, which can be attributed in part to sophisticated trauma management systems that more efficiently handle and record instances of polytrauma [6,38].

### Age Distribution

The age distribution of TBI patients in Ethiopia indicates that young adults, especially those aged 15–44, are significantly impacted. This observation aligns with similar findings in other low- and middle-income nations, where young adults face the greatest risk due to their engagement in outdoor activities, employment in physically intensive occupations, and heightened vulnerability to road traffic incidents [2]. Research conducted in Uganda and Tanzania has similarly revealed a significant prevalence of TBI among young adult males, primarily attributed to road traffic incidents and occupational risks [7,8,39]. This demographic trend is also observable in high-income nations; however, the causes of injury differ, with a greater proportion of TBIs arising from sports-related injuries and recreational activities instead of road traffic collisions [5,40].

### Gender Distribution

Our meta-analysis reveals that males constitute the majority of TBI cases in Ethiopia, reflecting a global trend where male patients generally account for 70% or more of TBI incidents [3]. This male predominance is particularly evident in contexts where road traffic injuries, violence, and workplace accidents are primary contributors to injury, as men are more often engaged in high-risk activities and professions. Research conducted in various African nations, including Ghana and Kenya, indicates comparable gender distributions. Conversely, the gender disparity observed in high-income countries is less pronounced, probably as a result of differing lifestyle habits and more effective injury prevention strategies [32,41–43].

### Urban-Rural Distribution

The distribution of TBI cases in Ethiopia reveals disparities, with rural populations experiencing higher incidence rates. Individuals residing in rural areas are particularly vulnerable to risks such as falls, violence and injuries related to animals, which are prevalent in agrarian lifestyles. Similar studies conducted in rural regions of India and Nepal indicate comparable trends, where rural TBIs frequently result from falls from heights, agricultural accidents, and restricted access to healthcare services [44].

### Causes of TBI

Assault stands out as a leading cause of TBI in Ethiopia, representing 36% of cases identified in this meta-analysis. This percentage is considerably greater than the statistics observed in numerous high-income nations, where assaults make up a lesser share of TBI incidents. For instance, in the United States, assaults are responsible for around 10% of TBI cases [5]. The elevated rate of TBIs in Ethiopia may be indicative of various socio-economic factors and increased instances of interpersonal violence, which are frequently associated with urbanization, poverty, and inadequate law enforcement resources. Similarly, other African nations, such as South Africa, exhibit comparable patterns, where interpersonal violence and assaults significantly contribute to TBIs, largely due to socioeconomic inequalities and elevated crime rates [2,3,45]

Road traffic incidents represent 35% of TBI cases in Ethiopia, a statistic that aligns with trends observed in other low- and middle-income nations, where road safety remains an increasing concern. This high prevalence can be linked to several factors, including rapid urbanization, inadequate infrastructure, and weak enforcement of traffic laws [31]. In Africa, RTIs rank among the primary causes of TBIs. Research conducted in Nigeria and Kenya highlights a significant impact from RTIs, largely attributed to the rising number of vehicles on the roads and insufficient road safety measures [46–48]. Conversely, high-income nations experience lower incidences of RTI-related TBIs, which can be partly explained by more rigorous enforcement of road safety regulations and the presence of effective emergency response services [5].

Falls account for 21% of TBI cases in Ethiopia, a statistic that aligns with the global mid-range. In high-income nations, falls are the primary cause of TBI, particularly among older adults, attributed to an aging demographic and a reduced incidence of injuries related to violence [49]. In Ethiopia, the rate may indicate the occupational hazards associated with the agrarian lifestyle of rural communities, where falls from heights, particularly during farming activities, are frequent. Research conducted in other low-resource environments, such as rural India and Nepal, reveals comparable patterns, with falls from trees and rooftops significantly contributing to cases of TBI among rural populations [44,50].

The remaining 7% of TBI cases in Ethiopia arise from less prevalent causes, such as injuries related to animals, falling objects, firearms, and accidents occurring in the workplace. These incidents are often linked to particular occupational hazards and lifestyle factors that are characteristic of rural Ethiopian and broader African communities. For example, animal-related TBIs are commonly documented in rural agrarian societies where there is frequent interaction with livestock [51]. In Ethiopia, firearm-related injuries occur less frequently than in nations with greater levels of firearm ownership, like the United States, where gunshot wounds significantly contribute to cases of TBI [52].

The distribution of GCS scores in our study (57% with scores of 13–15, 25% with scores of 9–12, and 18% with scores of 3–8) provides insight into the clinical presentation of TBI patients in Ethiopia. However, it is important to note that GCS scores were not used to classify TBI severity, as they can be influenced by factors such as intoxication, sedation, or systemic conditions. This aligns with the global trend observed in high-income countries, where mild TBIs comprise 70–90% of reported cases [5]. In LMICs, mild TBI cases may be underreported because of restricted access to healthcare and diagnostic facilities, indicating that the true prevalence could be greater. Our analysis found that moderate TBIs accounted for 25% of cases, which is somewhat higher than the rates typically seen in many high-income countries, where moderate TBI cases generally fall between 10% and 20% [53]. In Ethiopia, the increased severity of cases that could typically be classified as mild may be influenced by factors such as postponed medical intervention and a scarcity of neurosurgical services. Patients with GCS between 9–12 represented 18% of the overall total, which aligns with the typical range observed in LMICs. This percentage is somewhat higher than that found in high-income countries, where severe TBIs account for approximately 10% of cases [54]. Research conducted in various African nations has indicated comparable rates of severe TBI, linking these incidents to infrastructural issues and delays in response times [33].

The 17% prevalence of complications related to TBI observed in this analysis is consistent with findings from other LMICs, where similar rates have been reported. For example, research conducted in Uganda and Tanzania indicates that TBI patients experience complication rates ranging from 15% to 20%. Common issues include posttraumatic seizures, infections, and pneumonia, which are particularly prevalent due to limited access to intensive monitoring and specialized medical care [13,55,56]. In high-income nations, the rates of complications are generally lower, falling between 10% and 15%. This reduction can be attributed to the availability of advanced trauma care, regular preventive measures, and thorough infection control protocols [5].

The surgical intervention rate of 31% identified in this meta-analysis reflects the proportion of TBI cases where surgery was clinically indicated, rather than a direct measure of injury severity. Surgical intervention is typically warranted in cases with specific complications, such as epidural or subdural hematomas, midline shift, or elevated intracranial pressure. However, it is important to note that some severe TBI cases may not benefit from surgery, while milder cases with specific indications may require surgical management. The observed heterogeneity in surgical intervention rates (I² = 98.79%) may be attributed to differences in clinical practices, resource availability, and patient populations across studies. For example, limited access to diagnostic tools (e.g., CT scans) or neurosurgical expertise in rural areas may result in lower surgical intervention rates, despite the presence of severe injuries. This figure aligns with data from other LMICs, where surgery rates for TBI vary between 25% and 35%, influenced by the severity of the injuries and the healthcare resources available [2]. The demand for surgical procedures in Ethiopia is likely indicative of a greater prevalence of severe injuries that require surgical intervention, including hematoma evacuation or decompressive craniectomy. Nonetheless, in environments with constrained neurosurgical resources, delays in receiving surgical care may lead to deteriorated patient outcomes and a rise in complications.

Finally, this meta-analysis revealed an overall mortality rate of 12%, which falls within the range noted in other LMICs, although it is higher than the rates generally seen in high-income nations. In Sub-Saharan Africa, TBI mortality rates vary widely due to differences in healthcare access and trauma care resources, commonly reported between 10% and 15%. This aligns with observations from Uganda and Nigeria, where insufficient trauma care contributes to elevated early mortality rates [3]. In high-income environments, mortality rates are generally significantly lower, approximately 5–7%, due to the availability of advanced prehospital care, prompt interventions, and thorough post-injury monitoring [53]. The increased mortality observed in Ethiopia may be attributed to delays in seeking medical attention, a shortage of trained neurotrauma personnel, and insufficient intensive care resources, all of which hinder effective monitoring and management of severe cases.

### Implications of the study

Our findings have several implications for Ethiopian health policy and trauma care systems. The high prevalence of traumatic brain injury caused by road traffic accidents underscores the urgent need for targeted prevention strategies. These could include enforcing road safety regulations, improving driver education programs, and developing infrastructure such as pedestrian walkways and speed-reducing measures in high-risk areas. Additionally, the significant contribution of violence to traumatic brain injury highlights the necessity for community-based interventions aimed at reducing interpersonal conflicts and domestic violence.

Resource allocation should prioritize strengthening trauma care systems, including improving access to neurosurgical services, equipping healthcare facilities in underserved regions, and training healthcare professionals in the management of head injuries. Enhancing pre-hospital care systems, such as ambulance services and first responder training, is critical to reducing delays in care and improving outcomes for traumatic brain injury patients. Furthermore, public health campaigns can play a pivotal role in raising awareness about risk factors and preventive measures, contributing to long-term reductions in traumatic brain injury incidence.

By addressing these aspects, policymakers can develop a more comprehensive and equitable approach to managing and preventing traumatic brain injuries in Ethiopia.

## Conclusion

This meta-analysis examines TBI in Ethiopia, offering important insights into the prevalence, complications, and mortality rates linked to TBI within a low-resource context. The primary causes identified include assaults, road traffic accidents, and falls, with mild TBI being the most frequently occurring severity level. Complications occurred in around 17% of TBI cases, with prevalent issues such as posttraumatic seizures, aspiration pneumonia, and wound infections. Furthermore, close to one-third of TBI patients needed surgical procedures. The combined mortality rate of 12% underscores the considerable risk linked to TBI in Ethiopia, a situation likely worsened by insufficient access to prompt trauma care and neurosurgical facilities.

### Limitations of the study

- The most significant limitation of this study is the substantial heterogeneity (I² > 90%) observed across the included studies. This variability, driven by differences in regional settings, study designs, and data collection methods, may affect the generalizability and reliability of the pooled estimates. For example, the high heterogeneity in prevalence estimates (I² = 97%) suggests that the true burden of TBI in Ethiopia may vary significantly across regions and populations. Additionally, the uneven distribution of studies, with a concentration in urban areas such as Addis Ababa, may further limit the representativeness of the findings.
- Only published studies were included, which may introduce publication bias, as studies with significant findings are more likely to be published
- Many studies report TBI without access to advanced imaging modalities like CT or MRI, especially in rural settings. This lack of diagnostic resources may result in underreporting of TBI severity and complexity, as milder cases or non-obvious brain injuries could go undiagnosed
- The trauma care availability may vary across Ethiopia, and this variability is not accounted for in each study, which may skew data on outcomes, especially mortality and complications

### Recommendations

- Address Heterogeneity in Future Studies: Future research should aim to reduce heterogeneity by using standardized data collection methods, stratified sampling techniques, and subgroup analyses based on region, study design, and population characteristics. This will improve the reliability and generalizability of the findings.
- Conduct large-scale, nationally representative studies to capture a more accurate picture of TBI prevalence, causes, and outcomes across all Ethiopian regions, especially underserved rural areas
- Studies should investigate the effectiveness of preventive interventions, such as road safety initiatives, violence prevention programs, and occupational safety measures.
- Research on the capacity of hospitals and trauma centers to manage TBI cases, especially in rural and under-resourced areas
- Future studies could investigate the socioeconomic and cultural factors contributing to high TBI rates due to assaults, RTIs, and falls
- Encourage stratified sampling techniques to ensure proportional representation of regions with varying levels of healthcare access.
- Promote collaboration with local healthcare providers and community organizations to facilitate data collection in underserved and rural areas.
- We propose that future studies include; conference proceedings, registered trial databases such as clinicaltrials.gov., grey literature sources, such as theses or institutional reports, which may provide valuable insights from regions currently underrepresented in published research.
- Capacity Building: Providing training and resources for healthcare workers in rural and underserved areas to enhance their ability to identify and document TBI cases using available tools.
- Mobile Diagnostic Units: Advocating for the implementation of mobile diagnostic units equipped with portable imaging technologies, such as portable CT or point-of-care ultrasound, to improve diagnostic coverage in remote areas.
- Resource Allocation: Encouraging policymakers and stakeholders to prioritize the allocation of resources to expand access to advanced imaging modalities in regional and rural hospitals.
- Emphasizing the need for future studies to collect and incorporate data on trauma care availability, such as the number of trained healthcare providers, the presence of critical care facilities, and the accessibility of emergency medical services.
- Proposing that future research stratify analyses based on regional healthcare resources to account for disparities in trauma care availability.
- Recommending collaborations with regional health authorities to better understand and quantify the influence of trauma care variability on TBI outcomes.

## Supporting information

S1 Fig Contribution of road traffic incidents to traumatic brain injury cases in Ethiopia. The forest plot presents the pooled estimate and confidence intervals for road traffic incidents as a cause of TBI, highlighting variability across the included studies (n = 7854).
(DOCX)

S2 Fig Proportion of traumatic brain injuries caused by assault/violence in Ethiopia. This forest plot illustrates the pooled estimate of cases attributed to assault/violence, along with the confidence intervals from individual studies included in the meta-analysis (n = 7854).
(DOCX)

S3 Fig Prevalence of falls as a cause of traumatic brain injury in Ethiopia. This forest plot summarizes the pooled estimate for falls and their contribution to TBI cases, including confidence intervals and inter-study variability (n = 7854).
(DOCX)

S4 Fig Prevalence of other causes as a cause of traumatic brain injury in Ethiopia. This forest plot summarizes the pooled estimate for other causes and their contribution to TBI cases, including confidence intervals and inter-study variability (n = 7854).
(DOCX)

S5 Fig Forest plot showing male proportion in the included studies. (DOCX)

S6 Fig Forest plot showing female proportion in the included studies. (DOCX)

S7 Fig Forest plot showing patient proportion from rural areas in the included studies. (DOCX)

S8 Fig Forest plot showing patient proportion from urban areas in the included studies. (DOCX)

S9 Fig Forest plot showing adult patient proportion in the included studies. (DOCX)

S10 Fig Forest plot showing pediatric patient proportion in the included studies. (DOCX)

S11 Fig Forest plot showing proportion of mild head injury. (DOCX)

S12 Fig Forest plot showing proportion of moderate head injury. (DOCX)

S13 Fig Forest plot showing proportion of severe head injury. (DOCX)

S14 Fig Forest plot showing proportion of isolated TBI. (DOCX)

S15 Fig Forest plot showing proportion of polytrauma. (DOCX)

S16 Fig Forest plot showing proportion of contusion as an imaging finding. (DOCX)

S17 Fig Forest plot showing proportion of skull fracture as an imaging finding. (DOCX)

S18 Fig Forest plot showing proportion of epidural hematoma as an imaging finding. (DOCX)

S19 Fig Forest plot showing proportion of intracranial hemorrhage other than epidural hematoma as an imaging finding. (DOCX)

S20 Fig Forest plot showing mortality rate in the included studies. (DOCX)

S21 Fig Forest plot showing the prevalence of postraumatic seizure in the included studies. (DOCX)

S22 Fig Forest plot showing prevalence of aspiration pneumonia in the included studies. (DOCX)

S23 Fig Forest plot showing operation rate in the included studies. (DOCX)

S24 Fig Forest plot showing studies that report overall complication rate among TBI patients. (DOCX)

S25 Fig Funnel plot showing possible publication bias. (DOCX)

S26 Fig Mortality rate associated with traumatic brain injury in Ethiopia. This forest plot shows the pooled mortality rate and confidence intervals, based on data from the included studies. The analysis reveals regional variations and high heterogeneity among studies (n = 6885).
(DOCX)

S27 Fig Egger test for assessing publication bias for the prevalence and causes of traumatic brain injury (TBI). (DOCX)

S1 Table The studies excluded after full text review in the systematic review with their reasons of exclusion. (DOCX)

S2 Table A table showing all data extracted from the primary research sources for the systematic review. (DOCX)

S3 Table The result of quality assessment of included studies using Joanna Briggs Institute (JBI). (DOCX)

S4 Table Assessment of Certainty of Evidence Using the GRADE Approach. (DOCX)

## Abbreviations:

ED: Emergency department
LMIC: Low- and middle-income countries
PRISMA: Preferred Reporting Items for Systematic Reviews and Meta-Analyses
RTI: Road traffic accident
TBI: Traumatic brain injury.

## Declaration

MeSH terms

• Brain Injuries, Traumatic/ epidemiology*

• Ethiopia/ epidemiology,

• accidents,

• traffic, assault, fall-down, head injuries,/statistics and numerical data

• Female

• Humans

• Incidence

• Male

• Adult

• Pediatrics

• Outcome
