# Supplementary material for: Epidemiology of Traumatic brain injury in Ethiopia: A systematic review and meta-analysis of prevalence, mechanisms, and outcomes
Source: PLoS One. 2025 May 30;20(5):e0322641. doi: 10.1371/journal.pone.0322641 (PMC12124570; doi:10.1371/journal.pone.0322641)
Supplement: S1 Fig — The forest plot presents the pooled estimate and confidence intervals for road traffic incidents as a cause of TBI, highlighting variability across the included studies (n = 7854). (DOCX) [file pone.0322641.s001.docx]

Figure 1: Contribution of road traffic incidents to traumatic brain injury cases in Ethiopia. The forest plot presents the pooled estimate and confidence intervals for road traffic incidents as a cause of TBI, highlighting variability across the included studies (n=7854).
