# Supplementary material for: Epidemiology of Traumatic brain injury in Ethiopia: A systematic review and meta-analysis of prevalence, mechanisms, and outcomes
Source: PLoS One. 2025 May 30;20(5):e0322641. doi: 10.1371/journal.pone.0322641 (PMC12124570; doi:10.1371/journal.pone.0322641)
Supplement: S2 Fig — This forest plot illustrates the pooled estimate of cases attributed to assault/violence, along with the confidence intervals from individual studies included in the meta-analysis (n = 7854). (DOCX) [file pone.0322641.s002.docx]

Figure 2: Proportion of traumatic brain injuries caused by assault/violence in Ethiopia. This forest plot illustrates the pooled estimate of cases attributed to assault/violence, along with the confidence intervals from individual studies included in the meta-analysis (n=7854).
