# Supplementary material for: Epidemiology of Traumatic brain injury in Ethiopia: A systematic review and meta-analysis of prevalence, mechanisms, and outcomes
Source: PLoS One. 2025 May 30;20(5):e0322641. doi: 10.1371/journal.pone.0322641 (PMC12124570; doi:10.1371/journal.pone.0322641)
Supplement: S3 Fig — This forest plot summarizes the pooled estimate for falls and their contribution to TBI cases, including confidence intervals and inter-study variability (n = 7854). (DOCX) [file pone.0322641.s003.docx]

Figure 3: Prevalence of falls as a cause of traumatic brain injury in Ethiopia. This forest plot summarizes the pooled estimate for falls and their contribution to TBI cases, including confidence intervals and inter-study variability (n=7854).
