# Supplementary material for: Epidemiology of Traumatic brain injury in Ethiopia: A systematic review and meta-analysis of prevalence, mechanisms, and outcomes
Source: PLoS One. 2025 May 30;20(5):e0322641. doi: 10.1371/journal.pone.0322641 (PMC12124570; doi:10.1371/journal.pone.0322641)
Supplement: S14 Fig — (DOCX) [file pone.0322641.s014.docx]

Figure 14: forest plot showing proportion of isolated TBI
