# Supplementary material for: Epidemiology of Traumatic brain injury in Ethiopia: A systematic review and meta-analysis of prevalence, mechanisms, and outcomes
Source: PLoS One. 2025 May 30;20(5):e0322641. doi: 10.1371/journal.pone.0322641 (PMC12124570; doi:10.1371/journal.pone.0322641)
Supplement: S25 Fig — (DOCX) [file pone.0322641.s025.docx]

Figure 25: funnel plot showing possible publication bias

Figure 26: funnel plot showing publication bias among the included studies
