# Supplementary material for: Epidemiology of Traumatic brain injury in Ethiopia: A systematic review and meta-analysis of prevalence, mechanisms, and outcomes
Source: PLoS One. 2025 May 30;20(5):e0322641. doi: 10.1371/journal.pone.0322641 (PMC12124570; doi:10.1371/journal.pone.0322641)
Supplement: S26 Fig — This forest plot shows the pooled mortality rate and confidence intervals, based on data from the included studies. The analysis reveals regional variations and high heterogeneity among studies (n = 6885). (DOCX) [file pone.0322641.s026.docx]

#

Fig 26: Mortality rate associated with traumatic brain injury in Ethiopia. This forest plot shows the pooled mortality rate and confidence intervals, based on data from the included studies. The analysis reveals regional variations and high heterogeneity among studies (n=6885).
