# Supplementary material for: Epidemiology of Traumatic brain injury in Ethiopia: A systematic review and meta-analysis of prevalence, mechanisms, and outcomes
Source: PLoS One. 2025 May 30;20(5):e0322641. doi: 10.1371/journal.pone.0322641 (PMC12124570; doi:10.1371/journal.pone.0322641)
Supplement: S1 Table — (DOCX) [file pone.0322641.s028.docx]

| **Column1** | **First Author** | **Publication Year** | **Outcome** | **Reason for exclusion** |
| --- | --- | --- | --- | --- |
| 1 | Abate et al | 2021 | Excluded | No relevant outcome for this study |
| 2 | Abawa et al | 2016 | Excluded | No relevant outcome for this study |
| 3 | abayneh et al | 2019 | Excluded | No relevant outcome for this study |
| 4 | Abdi et al | 2022 | Excluded | No relevant outcome for this study |
| 5 | Abdie et al | 2022 | Excluded | No relevant outcome for this study |
| 6 | Abebe et al | 2022 | Excluded | No relevant outcome for this study |
| 7 | Abebe et al | 2015 | Excluded | No relevant outcome for this study |
| 8 | Abebe T | 2015 | Excluded | No relevant outcome for this study |
| 9 | Abebe T et al | 2024 | Included |  |
| 10 | Abie et al | 2023 | Excluded | No relevant outcome for this study |
| 11 | Abraha | 2024 | Excluded | No relevant outcome for this study |
| 12 | Adal et al | 2022 | Excluded | No relevant outcome for this study |
| 13 | Adal O | 2024 | Excluded | No relevant outcome for this study |
| 14 | Adamu et al | 2019 | Excluded | No relevant outcome for this study |
| 15 | Adamu et al | 2019 | Excluded | No relevant outcome for this study |
| 16 | Addis NA | 2024 | Excluded | No relevant outcome for this study |
| 17 | addisu et al | 2024 | Excluded | No relevant outcome for this study |
| 18 | Adem et al | 2024 | Excluded | No relevant outcome for this study |
| 19 | Adem et al | 2001 | Excluded | No relevant outcome for this study |
| 20 | Adiss et al | 2024 | Excluded | No relevant outcome for this study |
| 21 | Admassie et al | 2009 | Excluded | No relevant outcome for this study |
| 22 | Admassu et al | 2018 | Excluded | No relevant outcome for this study |
| 23 | Adugna | 2024 | Excluded | No relevant outcome for this study |
| 24 | Aenderl I | 2014 | Included |  |
| 25 | aerts et al | 2017 | Excluded | No relevant outcome for this study |
| 26 | Afacho et al | 2024 | Excluded | No relevant outcome for this study |
| 27 | Afenigus et al | 2021 | Excluded | No relevant outcome for this study |
| 28 | Ahmed et al | 2024 | Excluded | No relevant outcome for this study |
| 29 | Ahmed et al | 2021 | Excluded | No relevant outcome for this study |
| 30 | Ajaj et’al | 2013 | Excluded | No relevant outcome for this study |
| 31 | Alem KD | 2019 | Excluded | No relevant outcome for this study |
| 32 | alemayehu et al | 2020 | Excluded | No relevant outcome for this study |
| 33 | alemayehu et al | 2021 | Excluded | No relevant outcome for this study |
| 34 | Alemayehu et al | 2023 | Excluded | No relevant outcome for this study |
| 35 | Alemu et al | 2023 | Excluded | No relevant outcome for this study |
| 36 | Ali et al | 2024 | Excluded | No relevant outcome for this study |
| 37 | Ali EH | 2023 | Excluded | No relevant outcome for this study |
| 38 | Aliye et al | 2013 | Excluded | No relevant outcome for this study |
| 39 | Aliye K | 2023 | Excluded | No relevant outcome for this study |
| 40 | Almu et al | 2006 | Excluded | No relevant outcome for this study |
| 41 | Altaye et al | 2022 | Excluded | No relevant outcome for this study |
| 42 | Altaye et al | 2022 | Excluded | No relevant outcome for this study |
| 43 | Amanuel et al | 2021 | Excluded | No relevant outcome for this study |
| 44 | Amara et al | 2012 | Excluded | No relevant outcome for this study |
| 45 | Amare et’al | 2021 | Excluded | No relevant outcome for this study |
| 46 | Amare et’al | 2008 | Excluded | No relevant outcome for this study |
| 47 | Amdeslasie et al | 2017 | Included |  |
| 48 | Amdeslasie F | 2016 | Excluded | No relevant outcome for this study |
| 49 | Amdessilassie et al | 2022 | Excluded | No relevant outcome for this study |
| 50 | Ametaj et al | 2021 | Excluded | No relevant outcome for this study |
| 51 | Amme et al | 2021 | Excluded | No relevant outcome for this study |
| 52 | Amme et al | 2023 | Excluded | No relevant outcome for this study |
| 53 | Andualem et al | 2022 | Excluded | No relevant outcome for this study |
| 54 | Aragago et’al | 2022 | Excluded | No relevant outcome for this study |
| 55 | araya et al | 2021 | Excluded | No relevant outcome for this study |
| 56 | Asegid et al | 2021 | Excluded | No relevant outcome for this study |
| 57 | Asfaw et al | 2021 | Excluded | No relevant outcome for this study |
| 58 | Ashebir et al | 2024 | Excluded | No relevant outcome for this study |
| 59 | asmamaw et al | 2023 | Excluded | No relevant outcome for this study |
| 60 | Asmamaw et’al | 2019 | Excluded | No relevant outcome for this study |
| 61 | Asmamaw Y | 2019 | Excluded | No relevant outcome for this study |
| 62 | Asnakew S | 2022 | Excluded | No relevant outcome for this study |
| 63 | Assaye et al | 2020 | Excluded | No relevant outcome for this study |
| 64 | assefa et al | 2015 | Excluded | No relevant outcome for this study |
| 65 | Assefa et al | 2021 | Excluded | No relevant outcome for this study |
| 66 | assefa et al | 2023 | Excluded | No relevant outcome for this study |
| 67 | Assele DD | 2021 | Included |  |
| 68 | Atalay YA | 2024 | Excluded | No relevant outcome for this study |
| 69 | Ayalew et al | 2018 | Excluded | No relevant outcome for this study |
| 70 | Ayana B | 2012 | Excluded | No relevant outcome for this study |
| 71 | Ayana et al | 2012 | Excluded | No relevant outcome for this study |
| 72 | Ayana et al | 2012 | Excluded | No relevant outcome for this study |
| 73 | ayano et al | 2023 | Excluded | No relevant outcome for this study |
| 74 | Ayehu et al | 2023 | Excluded | No relevant outcome for this study |
| 75 | Ayehualem et al | 2021 | Excluded | No relevant outcome for this study |
| 76 | ayele et al | 2021 | Excluded | No relevant outcome for this study |
| 77 | Ayele et al | 2017 | Excluded | No relevant outcome for this study |
| 78 | Ayele A | 2024 | Included |  |
| 79 | Ayele BA | 2022 | Excluded | No relevant outcome for this study |
| 80 | Ayele BA | 2021 | Excluded | No relevant outcome for this study |
| 81 | ayele et al | 2023 | Excluded | No relevant outcome for this study |
| 82 | Ayele et’al | 2017 | Excluded | No relevant outcome for this study |
| 83 | Ayele et’al | 2020 | Excluded | No relevant outcome for this study |
| 84 | Aynalem et al | 2020 | Excluded | No relevant outcome for this study |
| 85 | Azaw et al | 2024 | Excluded | No relevant outcome for this study |
| 86 | Azezh et al | 2023 | Excluded | No relevant outcome for this study |
| 87 | Azmeraw et al | 2024 | Excluded | No relevant outcome for this study |
| 88 | Azolov VV | 1995 | Excluded | No relevant outcome for this study |
| 89 | bacha et al | 2023 | Excluded | No relevant outcome for this study |
| 90 | Baode et al | 2022 | Excluded | No relevant outcome for this study |
| 91 | Baru A | 2019 | Excluded | No relevant outcome for this study |
| 92 | Basore et al | 2021 | Excluded | No relevant outcome for this study |
| 93 | Baye et’al | 2024 | Excluded | No relevant outcome for this study |
| 94 | Baye ND | 2024 | Excluded | No relevant outcome for this study |
| 95 | Bayera et al | 2024 | Excluded | No relevant outcome for this study |
| 96 | Bayleyegn et’al | 2024 | Excluded | No relevant outcome for this study |
| 97 | Bedada et al | 2021 | Excluded | No relevant outcome for this study |
| 98 | Bedaso A | 2018 | Excluded | No relevant outcome for this study |
| 99 | Bedaso et’al | 2018 | Excluded | No relevant outcome for this study |
| 100 | bedie et al | 2019 | Excluded | No relevant outcome for this study |
| 101 | Bedry et al | 2020 | Included |  |
| 102 | Behru et al | 2015 | Excluded | No relevant outcome for this study |
| 103 | Bekele D | 2021 | Excluded | No relevant outcome for this study |
| 104 | Bekele et al | 2022 | Excluded | No relevant outcome for this study |
| 105 | belay et al | 2020 | Excluded | No relevant outcome for this study |
| 106 | Bell et al | 2004 | Excluded | No relevant outcome for this study |
| 107 | Berhan et al | 2001 | Excluded | No relevant outcome for this study |
| 108 | Berhe et al | 2017 | Excluded | No relevant outcome for this study |
| 109 | Beyene et al | 2020 | Excluded | No relevant outcome for this study |
| 110 | bezabih et al | 2018 | Excluded | No relevant outcome for this study |
| 111 | Bihonegn et al | 2023 | Excluded | No relevant outcome for this study |
| 112 | Biluts et al | 2009 | Excluded | No relevant outcome for this study |
| 113 | Biluts et al | 2017 | Included |  |
| 114 | Biluts H | 2009 | Excluded | No relevant outcome for this study |
| 115 | Birhanu et al | 2016 | Excluded | No relevant outcome for this study |
| 116 | Birkenmeier G | 2016 | Excluded | No relevant outcome for this study |
| 117 | Biru et al | 2019 | Excluded | No relevant outcome for this study |
| 118 | Biruk et al | 2007 | Excluded | No relevant outcome for this study |
| 119 | Biruk et al | 2020 | Excluded | No relevant outcome for this study |
| 120 | Bogale et al | 2021 | Excluded | No relevant outcome for this study |
| 121 | Botelho et al | 2022 | Excluded | No relevant outcome for this study |
| 122 | Bradly | 2021 | Excluded | No relevant outcome for this study |
| 123 | Bradshaw CJ | 2018 | Excluded | No relevant outcome for this study |
| 124 | Buh et al | 2022 | Excluded | No relevant outcome for this study |
| 125 | Bulcha et al | 2005 | Excluded | No relevant outcome for this study |
| 126 | Bushen et al | 2024 | Excluded | No relevant outcome for this study |
| 127 | Cadotte et al | 2010 | Excluded | No relevant outcome for this study |
| 128 | Candelo et al | 2024 | Excluded | No relevant outcome for this study |
| 129 | Chawla et al | 2015 | Excluded | No relevant outcome for this study |
| 130 | chokoto et al | 2014 | Excluded | No relevant outcome for this study |
| 131 | chweya et al | 2023 | Excluded | No relevant outcome for this study |
| 132 | clapham et al | 1990 | Excluded | No relevant outcome for this study |
| 133 | Clark D | 2022 | Excluded | No relevant outcome for this study |
| 134 | Crowe CS | 2020 | Excluded | No relevant outcome for this study |
| 135 | Daba et al | 2024 | Excluded | No relevant outcome for this study |
| 136 | Dagnaw Y et al | 2022 | Excluded | No relevant outcome for this study |
| 137 | Dawit et al | 2024 | Excluded | No relevant outcome for this study |
| 138 | de Berker HT | 2022 | Excluded | No relevant outcome for this study |
| 139 | de Berker HT | 2021 | Excluded | No relevant outcome for this study |
| 140 | de Oliveira AJM | 2021 | Excluded | No relevant outcome for this study |
| 141 | Degefa HG | 2024 | Excluded | No relevant outcome for this study |
| 142 | Degnet et al | 2021 | Excluded | No relevant outcome for this study |
| 143 | Deguale et al | 2024 | Excluded | No relevant outcome for this study |
| 144 | demamaw et al | 2011 | Excluded | No relevant outcome for this study |
| 145 | Demass et al | 2023 | Excluded | No relevant outcome for this study |
| 146 | Demass TB | 2023 | Excluded | No relevant outcome for this study |
| 147 | Demeke E | 2022 | Excluded | No relevant outcome for this study |
| 148 | Demeke et al | 2022 | Excluded | No relevant outcome for this study |
| 149 | Demissie et al | 2018 | Excluded | No relevant outcome for this study |
| 150 | Demlie TA | 2023 | Included |  |
| 151 | Dengela et al | 2022 | Excluded | No relevant outcome for this study |
| 152 | dent et al | 2019 | Excluded | No relevant outcome for this study |
| 153 | Denu et al | 2021 | Excluded | No relevant outcome for this study |
| 154 | Denu et al | 2023 | Excluded | No relevant outcome for this study |
| 155 | denu et al | 2022 | Excluded | No relevant outcome for this study |
| 156 | Derese et al | 2020 | Excluded | No relevant outcome for this study |
| 157 | Denu ZA | 2021 | Excluded | No relevant outcome for this study |
| 158 | Deresse et al | 2015 | Excluded | No relevant outcome for this study |
| 159 | Dessie et al | 2009 | Excluded | No relevant outcome for this study |
| 160 | Dessie et al | 2024 | Excluded | No relevant outcome for this study |
| 161 | Desta et’al | 2014 | Excluded | No relevant outcome for this study |
| 162 | Dibera et al | 2024 | Included |  |
| 163 | Diop et al | 2003 | Excluded | No relevant outcome for this study |
| 164 | Dobe et al | 2024 | Excluded | No relevant outcome for this study |
| 165 | Eaton et al | 2017 | Excluded | No relevant outcome for this study |
| 166 | Edlmann E | 2020 | Excluded | No relevant outcome for this study |
| 167 | edlmann et al | 1986 | Excluded | No relevant outcome for this study |
| 168 | Elias et al | 2005 | Excluded | No relevant outcome for this study |
| 169 | Elias et al | 2021 | Excluded | No relevant outcome for this study |
| 170 | Endeshaw et al | 2023 | Excluded | No relevant outcome for this study |
| 171 | Endeshaw et al | 2023 | Excluded | No relevant outcome for this study |
| 172 | Erilkh et al | 2002 | Excluded | No relevant outcome for this study |
| 173 | Eshete et al | 2018 | Included |  |
| 174 | Eyassu et al | 2024 | Excluded | No relevant outcome for this study |
| 175 | Fako et al | 2020 | Excluded | No relevant outcome for this study |
| 176 | fanta et al | 2010 | Excluded | No relevant outcome for this study |
| 177 | Fasika et al | 2016 | Excluded | No relevant outcome for this study |
| 178 | Fell et al | 2013 | Excluded | No relevant outcome for this study |
| 179 | Fell et al | 2014 | Excluded | No relevant outcome for this study |
| 180 | Fell MJ | 2014 | Excluded | No relevant outcome for this study |
| 181 | Ferede et al | 2021 | Excluded | No relevant outcome for this study |
| 182 | Ferede et al | 2020 | Excluded | No relevant outcome for this study |
| 183 | Feyissa et al | 2023 | Excluded | No relevant outcome for this study |
| 184 | Fikadu et al | 2012 | Excluded | No relevant outcome for this study |
| 185 | Fikadu A | 2021 | Excluded | No relevant outcome for this study |
| 186 | Fikre | 2014 | Excluded | No relevant outcome for this study |
| 187 | Fink EL | 2018 | Excluded | No relevant outcome for this study |
| 188 | Firew et al | 2025 | Excluded | No relevant outcome for this study |
| 189 | Firrissa et al | 2023 | Excluded | No relevant outcome for this study |
| 190 | Fite et’al | 2019 | Excluded | No relevant outcome for this study |
| 191 | Frohlich et al | 1990 | Excluded | No relevant outcome for this study |
| 192 | Fröhlich et al | 1989 | Excluded | No relevant outcome for this study |
| 193 | G/Michael S | 2023 | Included |  |
| 194 | Gadisa et al | 2023 | Excluded | No relevant outcome for this study |
| 195 | Collaborator | 2021 | Excluded | No relevant outcome for this study |
| 196 | Gebreeziabher et al | 2022 | Excluded | No relevant outcome for this study |
| 197 | Gebressilassie et al | 2019 | Excluded | No relevant outcome for this study |
| 198 | Gebrewold et al | 2016 | Excluded | No relevant outcome for this study |
| 199 | Gedeno K | 2023 | Excluded | No relevant outcome for this study |
| 200 | Gelaw et al | 2022 | Excluded | No relevant outcome for this study |
| 201 | Gelaw et al | 2014 | Excluded | No relevant outcome for this study |
| 202 | Gemechu et’al | 2009 | Excluded | No relevant outcome for this study |
| 203 | Gemechu et’al | 2024 | Excluded | No relevant outcome for this study |
| 204 | Getabalew et’al | 2023 | Included |  |
| 205 | Getachew et’al | 2024 | Excluded | No relevant outcome for this study |
| 206 | getahun et al | 2021 | Excluded | No relevant outcome for this study |
| 207 | Getahun et al | 2015 | Excluded | No relevant outcome for this study |
| 208 | Getahun et al | 2021 | Excluded | No relevant outcome for this study |
| 209 | Getahun S | 2021 | Excluded | No relevant outcome for this study |
| 210 | Getnet et al | 2021 | Excluded | No relevant outcome for this study |
| 211 | Getnet et al | 2019 | Excluded | No relevant outcome for this study |
| 212 | Gidey et al | 2023 | Excluded | No relevant outcome for this study |
| 213 | gilman et al | 2016 | Excluded | No relevant outcome for this study |
| 214 | Girma et al | 2013 | Excluded | No relevant outcome for this study |
| 215 | Girmay et al | 2015 | Excluded | No relevant outcome for this study |
| 216 | Gizachew et al | 2021 | Excluded | No relevant outcome for this study |
| 217 | Gezahagn et al | 2019 | Included |  |
| 218 | Global Burden of Disease 2016 Injury Collaborators | 2018 | Excluded | No relevant outcome for this study |
| 219 | Global Burden of Disease Cancer Collaboration | 2017 | Excluded | No relevant outcome for this study |
| 220 | gobeze et al | 2016 | Excluded | No relevant outcome for this study |
| 221 | Goshu et al | 2024 | Excluded | No relevant outcome for this study |
| 222 | Gudu W | 2024 | Excluded | No relevant outcome for this study |
| 223 | Gugssa et al | 2020 | Excluded | No relevant outcome for this study |
| 224 | Habtamu et al | 2023 | Excluded | No relevant outcome for this study |
| 225 | Habtamu et al | 2015 | Excluded | No relevant outcome for this study |
| 226 | Habtamu et al | 2019 | Excluded | No relevant outcome for this study |
| 227 | Habtamu et al | 2022 | Excluded | No relevant outcome for this study |
| 228 | Habte et’al | 2023 | Excluded | No relevant outcome for this study |
| 229 | Habte YW | 2024 | Excluded | No relevant outcome for this study |
| 230 | Habte YW et al | 2023 | Excluded | No relevant outcome for this study |
| 231 | haftu et al | 2018 | Excluded | No relevant outcome for this study |
| 232 | Hagmann et al | 2014 | Excluded | No relevant outcome for this study |
| 233 | Hagos A | 2022 | Included |  |
| 234 | Hagos et al | 2015 | Excluded | No relevant outcome for this study |
| 235 | Haile et al | 2015 | Excluded | No relevant outcome for this study |
| 236 | Hailu et al | 2023 | Excluded | No relevant outcome for this study |
| 237 | Hailu et al | 2022 | Excluded | No relevant outcome for this study |
| 238 | Hailu et al | 2022 | Excluded | No relevant outcome for this study |
| 239 | Hampton T et al | 2017 | Excluded | No relevant outcome for this study |
| 240 | Harris et al | 2018 | Excluded | No relevant outcome for this study |
| 241 | Hassan et al | 2022 | Excluded | No relevant outcome for this study |
| 242 | Honeyman C | 2020 | Excluded | No relevant outcome for this study |
| 243 | Honeyman et al | 2020 | Excluded | No relevant outcome for this study |
| 244 | Hubena et al | 2018 | Excluded | No relevant outcome for this study |
| 245 | Huijing MA | 2011 | Excluded | No relevant outcome for this study |
| 246 | Hulin et al | 1982 | Excluded | No relevant outcome for this study |
| 247 | Hunchak et al | 2015 | Excluded | No relevant outcome for this study |
| 248 | Hunchak C | 2015 | Excluded | No relevant outcome for this study |
| 249 | Hunchat et al | 2015 | Excluded | No relevant outcome for this study |
| 250 | Hussein AA | 2022 | Excluded | No relevant outcome for this study |
| 251 | Hutchinson PJ | 2019 | Excluded | No relevant outcome for this study |
| 252 | hzkel et al | 2021 | Excluded | No relevant outcome for this study |
| 253 | Jiang et al | 2012 | Excluded | No relevant outcome for this study |
| 254 | Joannides AJ | 2024 | Excluded | No relevant outcome for this study |
| 255 | Jones et al | 2022 | Excluded | No relevant outcome for this study |
| 256 | Jong et al | 2015 | Excluded | No relevant outcome for this study |
| 257 | Joseph M | 2023 | Excluded | No relevant outcome for this study |
| 258 | Joseph M | 2023 | Excluded | No relevant outcome for this study |
| 259 | Jovel et al | 2018 | Excluded | No relevant outcome for this study |
| 260 | kaplan et al | 2009 | Excluded | No relevant outcome for this study |
| 261 | kasa et al | 2023 | Excluded | No relevant outcome for this study |
| 262 | Kebede et al | 2024 | Excluded | No relevant outcome for this study |
| 263 | kecec et al | 2008 | Excluded | No relevant outcome for this study |
| 264 | Kefale et al | 2020 | Excluded | No relevant outcome for this study |
| 265 | Kegnu et al | 2018 | Excluded | No relevant outcome for this study |
| 266 | Kejela et al | 2024 | Excluded | No relevant outcome for this study |
| 267 | kempf et al | 2012 | Excluded | No relevant outcome for this study |
| 268 | Ketama et al | 2015 | Excluded | No relevant outcome for this study |
| 269 | Kibret YT | 2024 | Excluded | No relevant outcome for this study |
| 270 | kidanu et al | 2011 | Excluded | No relevant outcome for this study |
| 271 | kifle et al | 2022 | Excluded | No relevant outcome for this study |
| 272 | Kim et al | 2020 | Excluded | No relevant outcome for this study |
| 273 | Kinori M | 2013 | Excluded | No relevant outcome for this study |
| 274 | Kloos et al | 1992 | Excluded | No relevant outcome for this study |
| 275 | Krishnan Muthaiah et al | 2025 | Excluded | No relevant outcome for this study |
| 276 | krol et al | 2011 | Excluded | No relevant outcome for this study |
| 277 | Kumera et al | 2015 | Excluded | No relevant outcome for this study |
| 278 | Laeke et al | 2023 | Excluded | No relevant outcome for this study |
| 279 | Lelisa | 2021 | Excluded | No relevant outcome for this study |
| 280 | Laeke T | 2021 | Included |  |
| 281 | Laeke T | 2019 | Excluded | No relevant outcome for this study |
| 282 | Laeke T | 2021 | Excluded | No relevant outcome for this study |
| 283 | Lambru et al | 2021 | Excluded | No relevant outcome for this study |
| 284 | landes et al | 2015 | Excluded | No relevant outcome for this study |
| 285 | Landes M | 2017 | Included |  |
| 286 | Latebo AA | 2024 | Excluded | No relevant outcome for this study |
| 287 | Latoto et al | 2017 | Excluded | No relevant outcome for this study |
| 288 | Laytin et al | 2018 | Excluded | No relevant outcome for this study |
| 289 | leggess et al | 2022 | Excluded | No relevant outcome for this study |
| 290 | Lehre et al | 2015 | Excluded | No relevant outcome for this study |
| 291 | Lester et al | 1978 | Excluded | No relevant outcome for this study |
| 292 | Liu et al | 2023 | Excluded | No relevant outcome for this study |
| 293 | Lodamo et al | 2020 | Excluded | No relevant outcome for this study |
| 294 | Lola et al | 2019 | Excluded | No relevant outcome for this study |
| 295 | Lund-johnson et al | 2017 | Excluded | No relevant outcome for this study |
| 296 | lyons et al | 2022 | Excluded | No relevant outcome for this study |
| 297 | Macielak RJ | 2022 | Excluded | No relevant outcome for this study |
| 298 | Mahari et al | 2022 | Excluded | No relevant outcome for this study |
| 299 | Malakhov LI et al | 1992 | Excluded | No relevant outcome for this study |
| 300 | Mamo et al | 2023 | Excluded | No relevant outcome for this study |
| 301 | Mamo et al | 2023 | Excluded | No relevant outcome for this study |
| 302 | Manakunda et al | 2018 | Excluded | No relevant outcome for this study |
| 303 | Mariam AG et al | 1983 | Excluded | No relevant outcome for this study |
| 304 | mbaye et al | 2021 | Excluded | No relevant outcome for this study |
| 305 | Mehari ZA | 2014 | Excluded | No relevant outcome for this study |
| 306 | Mekonen et al | 2019 | Excluded | No relevant outcome for this study |
| 307 | Mekonen et al | 2021 | Excluded | No relevant outcome for this study |
| 308 | Mekuria et al | 2013 | Excluded | No relevant outcome for this study |
| 309 | melese et al | 2012 | Excluded | No relevant outcome for this study |
| 310 | melkamu et al | 2022 | Excluded | No relevant outcome for this study |
| 311 | Mengesha et al | 2022 | Excluded | No relevant outcome for this study |
| 312 | Mengesha et al | 2024 | Excluded | No relevant outcome for this study |
| 313 | Mengesha et al | 2020 | Excluded | No relevant outcome for this study |
| 314 | Mengesha et al | 2023 | Excluded | No relevant outcome for this study |
| 315 | Mengistu et al | 2021 | Excluded | No relevant outcome for this study |
| 316 | Mengistu et al | 2021 | Excluded | No relevant outcome for this study |
| 317 | Mengistu et al | 2012 | Excluded | No relevant outcome for this study |
| 318 | Mengistu Z | 2012 | Excluded | No relevant outcome for this study |
| 319 | Mengste et al | 2023 | Excluded | No relevant outcome for this study |
| 320 | meragiaw et al | 2016 | Excluded | No relevant outcome for this study |
| 321 | Mere et al | 2017 | Excluded | No relevant outcome for this study |
| 322 | Merrie et al | 2019 | Excluded | No relevant outcome for this study |
| 323 | Mersha A | 2020 | Excluded | No relevant outcome for this study |
| 324 | Mersha et al | 2021 | Excluded | No relevant outcome for this study |
| 325 | Messelu et al | 2024 | Excluded | No relevant outcome for this study |
| 326 | Mezemir et al | 2020 | Excluded | No relevant outcome for this study |
| 327 | Miftah et al | 2017 | Excluded | No relevant outcome for this study |
| 328 | Miskir et al | 2023 | Excluded | No relevant outcome for this study |
| 329 | Mohammed et al | 2017 | Excluded | No relevant outcome for this study |
| 330 | Mohan M | 2021 | Excluded | No relevant outcome for this study |
| 331 | Molla et’al | 2024 | Excluded | No relevant outcome for this study |
| 332 | Molla YD | 2024 | Excluded | No relevant outcome for this study |
| 333 | Molla YD | 2024 | Excluded | No relevant outcome for this study |
| 334 | Molla YD | 2023 | Excluded | No relevant outcome for this study |
| 335 | Molla YD | 2024 | Excluded | No relevant outcome for this study |
| 336 | Mossisa et al | 2021 | Excluded | No relevant outcome for this study |
| 337 | Muche et al | 2023 | Excluded | No relevant outcome for this study |
| 338 | Mulugeta et al | 2024 | Excluded | No relevant outcome for this study |
| 339 | Mulugeta et al | 2015 | Excluded | No relevant outcome for this study |
| 340 | Mulugeta et’al | 2024 | Excluded | No relevant outcome for this study |
| 341 | Mulugeta H | 2024 | Excluded | No relevant outcome for this study |
| 342 | Mussa et al | 2021 | Excluded | No relevant outcome for this study |
| 343 | nasir et al | 2022 | Excluded | No relevant outcome for this study |
| 344 | Nega et al | 2002 | Excluded | No relevant outcome for this study |
| 345 | Negese et al | 2017 | Excluded | No relevant outcome for this study |
| 346 | nida et al | 2008 | Excluded | No relevant outcome for this study |
| 347 | NIGATU et al | 2022 | Excluded | No relevant outcome for this study |
| 348 | Niggussie et al | 2008 | Excluded | No relevant outcome for this study |
| 349 | Nigussie et al | 2021 | Excluded | No relevant outcome for this study |
| 350 | Nordanger et al | 2007 | Excluded | No relevant outcome for this study |
| 351 | Nunn TR | 2018 | Excluded | No relevant outcome for this study |
| 352 | ogada et al | 2019 | Excluded | No relevant outcome for this study |
| 353 | Osakwe et al | 2001 | Excluded | No relevant outcome for this study |
| 354 | oumer et al | 2020 | Excluded | No relevant outcome for this study |
| 355 | Pappadis et al | 2011 | Excluded | No relevant outcome for this study |
| 356 | Parker RK | 2020 | Excluded | No relevant outcome for this study |
| 357 | Perez KM | 2024 | Excluded | No relevant outcome for this study |
| 358 | Pital et al | 2016 | Excluded | No relevant outcome for this study |
| 359 | prodentale et al | 2011 | Excluded | No relevant outcome for this study |
| 360 | Rand et al | 2014 | Excluded | No relevant outcome for this study |
| 361 | Regasa et al | 2022 | Excluded | No relevant outcome for this study |
| 362 | Rhamni et al | 2016 | Excluded | No relevant outcome for this study |
| 363 | Richer et al | 2023 | Excluded | No relevant outcome for this study |
| 364 | RWIZA et al | 1992 | Excluded | No relevant outcome for this study |
| 365 | Sadik et al | 2019 | Excluded | No relevant outcome for this study |
| 366 | Sahlu A | 2020 | Excluded | No relevant outcome for this study |
| 367 | Sakolo et al | 2022 | Excluded | No relevant outcome for this study |
| 368 | Salia SM | 2018 | Excluded | No relevant outcome for this study |
| 369 | Santos et al | 2021 | Excluded | No relevant outcome for this study |
| 370 | Sartelli M | 2022 | Excluded | No relevant outcome for this study |
| 371 | Schierer et al | 2019 | Excluded | No relevant outcome for this study |
| 372 | schreiber et al | 1995 | Excluded | No relevant outcome for this study |
| 373 | Seadi et al | 2021 | Excluded | No relevant outcome for this study |
| 374 | Seid et’al | 2015 | Excluded | No relevant outcome for this study |
| 375 | Senay et al | 2019 | Excluded | No relevant outcome for this study |
| 376 | Sete G | 2025 | Excluded | No relevant outcome for this study |
| 377 | Sewagegn et al | 2017 | Excluded | No relevant outcome for this study |
| 378 | Shiferaw et’al | 2022 | Excluded | No relevant outcome for this study |
| 379 | Shiferaw MY | 2022 | Excluded | No relevant outcome for this study |
| 380 | Shitahun et al | 2020 | Excluded | No relevant outcome for this study |
| 381 | Shoberian et al | 2021 | Excluded | No relevant outcome for this study |
| 382 | Sirahbizu et al | 2018 | Excluded | No relevant outcome for this study |
| 383 | Sitot et al | 2022 | Excluded | No relevant outcome for this study |
| 384 | Smith et al | 2013 | Excluded | No relevant outcome for this study |
| 385 | Smith ZA | 2013 | Excluded | No relevant outcome for this study |
| 386 | sohier et al | 1999 | Excluded | No relevant outcome for this study |
| 387 | solano et al | 2017 | Excluded | No relevant outcome for this study |
| 388 | solomon et al | 2018 | Excluded | No relevant outcome for this study |
| 389 | Solomon et al | 2020 | Excluded | No relevant outcome for this study |
| 390 | Stachura et al | 2017 | Excluded | No relevant outcome for this study |
| 391 | Starr N | 2024 | Excluded | No relevant outcome for this study |
| 392 | sultan et al | 2018 | Excluded | No relevant outcome for this study |
| 393 | sultan et al | 2024 | Excluded | No relevant outcome for this study |
| 394 | Sultan M | 2024 | Excluded | No relevant outcome for this study |
| 395 | Sultan M | 2018 | Excluded | No relevant outcome for this study |
| 396 | Tadesse et al | 2014 | Excluded | No relevant outcome for this study |
| 397 | Tadesse et al | 2015 | Excluded | No relevant outcome for this study |
| 398 | taklehaymanot et al | 1990 | Excluded | No relevant outcome for this study |
| 399 | Tanne et al | 2008 | Excluded | No relevant outcome for this study |
| 400 | tarekegn et al | 2016 | Excluded | No relevant outcome for this study |
| 401 | Taye et al | 2022 | Excluded | No relevant outcome for this study |
| 402 | Techane et al | 2024 | Excluded | No relevant outcome for this study |
| 403 | Tedla et al | 2024 | Excluded | No relevant outcome for this study |
| 404 | Tefera EA | 2023 | Excluded | No relevant outcome for this study |
| 405 | Tegegne NG | 2023 | Excluded | No relevant outcome for this study |
| 406 | Tekle et al | 2015 | Excluded | No relevant outcome for this study |
| 407 | Tekle-Haimanot R | 1997 | Excluded | No relevant outcome for this study |
| 408 | Temesgen et al | 2019 | Excluded | No relevant outcome for this study |
| 409 | Tenkir A | 2010 | Excluded | No relevant outcome for this study |
| 410 | Terefe et al | 2001 | Excluded | No relevant outcome for this study |
| 411 | Tesahye et al | 2017 | Excluded | No relevant outcome for this study |
| 412 | Tesfahun et al | 2024 | Excluded | No relevant outcome for this study |
| 413 | Tesfaye et al | 2008 | Excluded | No relevant outcome for this study |
| 414 | Tesfaw et al | 2021 | Included |  |
| 415 | Teshita et’al | 2024 | Excluded | No relevant outcome for this study |
| 416 | Teshita G | 2023 | Excluded | No relevant outcome for this study |
| 417 | Teshome A | 2017 | Excluded | No relevant outcome for this study |
| 418 | Teshome AA | 2022 | Excluded | No relevant outcome for this study |
| 419 | Teshome et’al | 2022 | Excluded | No relevant outcome for this study |
| 420 | Teshome et’al | 2004 | Excluded | No relevant outcome for this study |
| 421 | Tewabe et al | 2024 | Excluded | No relevant outcome for this study |
| 422 | Tibebu et al | 2023 | Excluded | No relevant outcome for this study |
| 423 | Tigeneh et al | 2015 | Excluded | No relevant outcome for this study |
| 424 | Tilahun et al | 2022 | Excluded | No relevant outcome for this study |
| 425 | Tilahun et al | 2017 | Excluded | No relevant outcome for this study |
| 426 | Tilahun et al | 2024 | Excluded | No relevant outcome for this study |
| 427 | Tilahun L | 2024 | Excluded | No relevant outcome for this study |
| 428 | Tirukelem | 2021 | Excluded | No relevant outcome for this study |
| 429 | Tiruneh et’al | 2014 | Excluded | No relevant outcome for this study |
| 430 | Tiruneh et’al | 2022 | Excluded | No relevant outcome for this study |
| 431 | Tiwari A | 2021 | Excluded | No relevant outcome for this study |
| 432 | tokdimer et al | 2009 | Excluded | No relevant outcome for this study |
| 433 | Tolosa et al | 2023 | Excluded | No relevant outcome for this study |
| 434 | tomkins et al | 2011 | Excluded | No relevant outcome for this study |
| 435 | tsadik et al | 2020 | Excluded | No relevant outcome for this study |
| 436 | Veerappan VR | 2022 | Excluded | No relevant outcome for this study |
| 437 | walle et al | 2016 | Included |  |
| 438 | Weldehana et al | 2024 | Excluded | No relevant outcome for this study |
| 439 | Welu et al | 2023 | Excluded | No relevant outcome for this study |
| 440 | Werede et al | 2020 | Excluded | No relevant outcome for this study |
| 441 | winkeler et al | 2011 | Excluded | No relevant outcome for this study |
| 442 | Wirtz et al | 2013 | Excluded | No relevant outcome for this study |
| 443 | Wolde et al | 2019 | Excluded | No relevant outcome for this study |
| 444 | Wolde et al | 2008 | Excluded | No relevant outcome for this study |
| 445 | woldearagay et al | 2024 | Excluded | No relevant outcome for this study |
| 446 | woldegize et al | 2017 | Excluded | No relevant outcome for this study |
| 447 | Woldehawariat et al | 2019 | Excluded | No relevant outcome for this study |
| 448 | Woldemeskel et al | 2017 | Excluded | No relevant outcome for this study |
| 449 | Woldemichael et al | 2011 | Excluded | No relevant outcome for this study |
| 450 | Woldesenbet et’al | 2024 | Excluded | No relevant outcome for this study |
| 451 | Woldesillasie et al | 2020 | Excluded | No relevant outcome for this study |
| 452 | Woldu et al | 2017 | Excluded | No relevant outcome for this study |
| 453 | Wondimu et al | 2007 | Excluded | No relevant outcome for this study |
| 454 | Wondmu et al | 2018 | Excluded | No relevant outcome for this study |
| 455 | Worku et al | 2013 | Excluded | No relevant outcome for this study |
| 456 | Worku et al | 2022 | Excluded | No relevant outcome for this study |
| 457 | Worku et al | 2002 | Excluded | No relevant outcome for this study |
| 458 | Worku et al | 2022 | Excluded | No relevant outcome for this study |
| 459 | wright et al | 2016 | Excluded | No relevant outcome for this study |
| 460 | Wubete et al | 2020 | Excluded | No relevant outcome for this study |
| 461 | Wubetie et al | 2015 | Excluded | No relevant outcome for this study |
| 462 | Yasin YJ | 2022 | Excluded | No relevant outcome for this study |
| 463 | Yeakob et al | 2023 | Excluded | No relevant outcome for this study |
| 464 | Yemaneh et al | 2017 | Excluded | No relevant outcome for this study |
| 465 | Yesuf et al | 2016 | Excluded | No relevant outcome for this study |
| 466 | Yimam et al | 2022 | Excluded | No relevant outcome for this study |
| 467 | Yimam et’al | 2023 | Excluded | No relevant outcome for this study |
| 468 | Yimam EW | 2023 | Excluded | Case report |
| 469 | yohannis et al | 2021 | Excluded | No relevant outcome for this study |
| 470 | Yosha et al | 2021 | Excluded | No relevant outcome for this study |
| 471 | zarowsky et al | 2000 | Excluded | No relevant outcome for this study |
| 472 | Zekiel et al | 2020 | Excluded | No relevant outcome for this study |
| 473 | Zerihun N | 1993 | Excluded | No relevant outcome for this study |
| 474 | Zewdu M | 2024 | Excluded | No relevant outcome for this study |
| 475 | zewude et al | 2018 | Excluded | No relevant outcome for this study |
| 476 | zewude et al | 2022 | Excluded | No relevant outcome for this study |
| 477 | Zewudie et al | 2023 | Excluded | No relevant outcome for this study |
| 478 | Zewudu et’al | 2024 | Excluded | No relevant outcome for this study |

Table 1: the studies excluded after full text review in the systematic review with their reasons of exclusion
