# Supplementary material for: Epidemiology of Traumatic brain injury in Ethiopia: A systematic review and meta-analysis of prevalence, mechanisms, and outcomes
Source: PLoS One. 2025 May 30;20(5):e0322641. doi: 10.1371/journal.pone.0322641 (PMC12124570; doi:10.1371/journal.pone.0322641)
Supplement: S2 Table — (DOCX) [file pone.0322641.s029.docx]

| **Column1** | **Column2** | **Column3** | **Column4** | **Column5** | **Column6** | **Column7** | **Column8** | **Column9** | **Column10** | **Column11** | **Column12** | **Column13** | **Column14** | **Column15** | **Column16** | **Column17** | **Column18** | **Column19** | **Column20** | **Column21** | **Column22** | **Column23** | **Column24** | **Column25** | **Column26** | **Column27** | **Column28** | **Column29** | **Column30** | **Column31** |
| --- | --- | --- | --- | --- | --- | --- | --- | --- | --- | --- | --- | --- | --- | --- | --- | --- | --- | --- | --- | --- | --- | --- | --- | --- | --- | --- | --- | --- | --- | --- |
| **Study ID,year** | **Study Design** | **Region** | **Age** | **Sex-Male** | **Sex-Female** | **Rural** | **Urban** | **Adult** | **Pediatrics** | **Sample Size** | **Cause: Road Traffic Accidents** | **Cause: Falls** | **Cause: Interpersonal Violence** | **Cause: Occupational Hazards and others** | **Severity-mild** | **Severity-moderate** | **Severity-severe** | **Isolated TBI** | **Polytrauma** | **types of injury-contusion** | **types of injury-SF** | **Types of injury-EDH** | **Type of injury-ICH** | **Outcome: Mortality Rate** | **Length of Hospital Stay** | **complications-overall** | **Data Extractors** | **Date of data extraction** |  | **Article included in the study** |
| Abebe et al, 2024 | Retrospective cohort | Sidama | 35.82 | 70.9 | 29.1 | 27.4 | 72.6 | 954 | 10.1 | 1029 | 37.1 | 38.5 | 20 | 4.4 | 85.2 | 11.2 | 3.6 | 86.5 | 13.5 | 38.1 | 15.8 | 33.1 | 13 | 1.7 | 4.46 | 5.5 | YD and HT | August 15- September 24/2024 | | Yes |
| Aenderl et al, 2014 | prospective | oromia | 20 | 90 | 10 | NR | NR | 67 | 33 | 52 | 36.5 | 15.4 | 38.5 | 9.6 | 50 | 14 | 35 | 63 | 37 | NR | NR | NR | NR | 21.2 | NR | 12 | YD and HT | August 15- September 24/2024 | | Yes |
| Amdeslasie et al, 2017 | Retrospective cohort | Tigray | 25 | 79.7 | 20.3 | 43.7 | 56.3 | 75.6 | 24.4 | 750 | 24.9 | 41.7 | 24.8 | 4.9 | 62.1 | 24.2 | 13.6 | 45.9 | 54.1 | 10 | 19.8 | 3.3 | 0 | 8.3 | 7 | 11.1 | YD and HT | August 15- September 24/2024 | | Yes |
| Assele et al, 2021 | Retrospective cohort | Sidama | 26 | 83.43 | 16.57 | NR | NR | 77.9 | 22.1 | 1159 | 61.1 | 13.9 | 25 | 0 | 66.6 | 20.3 | 13.1 | 74.37 | 25.63 | 13.8 | 15.9 | 12.2 | 4.5 | 12.7 | 3 | 14.5 | YD and HT | August 15- September 24/2024 | | Yes |
| Ayele et al, 2024 | Retrospective cohort | Amhara | 34.9 | 77.4 | 22.6 | 81.1 | 18.9 | 100 | 0 | 429 | 44.1 | 12.8 | 38.9 | 0 | 11.7 | 53.1 | 35.2 | 23.3 | 76.7 | 8.1 | 52.6 | 35.4 | 14.2 | 33.8 | 7 | 20.7 | YD and HT | August 15- September 24/2024 | | Yes |
| Bedry et al, 2020 | cross-sectional | Sidama | 7.66 | 68.8 | 31.2 | 29 | 71 | 0 | 100 | 317 | 45.4 | 32.8 | 12.6 | 9.1 | 72.9 | 19.2 | 7.9 | 19.3 | 80.7 | 9.4 | 29.7 | 0.2 | 0.4 | 3.2 | 5 | 11.6 | YD and HT | August 15- September 24/2024 | | Yes |
| Biluts et al, 2017 | cross-sectional | AA | 32.3 | 95.6 | 4.4 | 68 | 23 | 81 | 10 | 91 | 8.8 | 11 | 75.8 | 4.4 | 37.4 | 29.7 | 32.9 | 76.2 | 23.8 | NR | NR | 86.8 | 1 | 18.7 | NR | 15.4 | YD and HT | August 15- September 24/2024 | | Yes |
| Demlie et al, 2023 | Retrospective cohort | Amhara | 32 | 85.6 | 14.4 | 65.7 | 34.3 | 544 | 0 | 544 | 25.3 | 13.8 | 60.1 | 0.74 | 54.2 | 30.5 | 15.3 | 69.1 | 30.1 | 22.9 |  | 11.4 | 6.4 | 13.2 | NR | 19 | YD and HT | August 15- September 24/2024 | | Yes |
| Dibera et al, 2024 | prospective | oromia | 29.8 | 72 | 28 | 40.6 | 59.4 | 175 | 0 | 175 | 58.9 | 10.9 | 26.8 | 3.4 | 60 | 32.6 | 7.43 | 71.2 | 28.8 | 10.3 | 33.1 | 13.7 | 7.43 | 12.6 | 5.66 | 31.4 | YD and HT | August 15- September 24/2024 | | Yes |
| Eshete et al, 2018 | Retrospective cohort | SERS | 24.7 | 71.7 | 28.3 | 55.7 | 44.3 | 70.8 | 29.2 | 106 | 44.1 | 19.8 | 33 | 7.5 | 52.8 | 15.1 | 32.1 | 83.1 | 16.9 | NR | 37.7 | NR | NR | 11.3 | 5 | 28.3 | YD and HT | August 15- September 24/2024 | | Yes |
| Tesfaw et al, 2021 | cross-sectional | Amhara | 41.4 | 71.6 | 28.4 | 70 | 30 | 370 | 0 | 370 | 24.8 | 31.4 | 43.8 | 0 | 89.2 | 6.5 | 4.3 | 31 | 69 | NR | NR | NR | NR | 9.5 | 4 | 4.3 | YD and HT | August 15- September 24/2024 | | Yes |
| Getabalew et al, 2023 | retrospective cross | Amhara | 9.2 | 65.3 | 34.7 | 62.1 | 37.9 | 0 | 404 | 404 | 24 | 47 | 12.6 | 16.3 | 54.7 | 36.4 | 8.9 | 61.5 | 39.5 | 3.5 | 18 | 0 | 2.2 | 5.9 | 5 | 15.3 | YD and HT | August 15- September 24/2024 | | Yes |
| Gezahegn et al, 2019 | prospective | SNNE | 36.9 | 84.4 | 15.6 | 94.4 | 5.6 | 72 | 18 | 90 | 24.4 | 19 | 29 | 24.4 | 60 | 16.7 | 23.3 | 63.2 | 26.8 | NR | 4.3 | 5.4 | 7.2 | 10.8 | NR | 2.6 | YD and HT | August 15- September 24/2024 | | Yes |
| G/Michael et al, 2023 | cross-sectional | Amhara | 26 | 61.2 | 38.8 | 39.3 | 60.7 | 100 | 0 | 483 | 37.5 | 18.4 | 24.2 | 19.9 | 33.1 | 30.8 | 36 | 48.2 | 51.8 | 12.4 | 11.2 | 13.4 | 13.7 | 24.8 | 6 | 29.9 | YD and HT | August 15- September 24/2024 | | Yes |
| Hagos et al, 2022 | cross-sectional | AA | 30.4 | 75 | 25 | 68.7 | 31.3 | 83.2 | 14.8 | 304 | 45.4 | 17.8 | 28.3 | 8.6 | 66.1 | 17 | 15.8 | 68.4 | 31.6 | 46 | 63.1 | 13.7 | 9.2 | 5.6 | 7 | 11.8 | YD and HT | August 15- September 24/2024 | | Yes |
| Landes et al, 2017 | prospective | AA | 26 | 86.8 | 13.2 | 0 | 204 | 95.6 | 4.4 | 204 | 41 | 12.7 | 40.7 | 4.9 | 56 | 19.1 | 23 | 74 | 26 | 21.7 | 41 | 18.1 | 6.3 | 10.3 | NR | NR | YD and HT | August 15- September 24/2024 | | Yes |
| Laeke et al, 2021 | cross-sectional | AA | 29 | 91.3 | 8.7 | 18.3 | 81.7 | 17.1 | 82.9 | 1087 | 15.8 | 8.1 | 69.9 | 6.1 | 52.1 | 29.1 | 15.5 | 96.9 | 3.1 | 1.4 | 28.6 | 27.7 | 3.9 | 8.2 | 4 | 17 | YD and HT | August 15- September 24/2024 | | Yes |
| Walle et al, 2016 | cross-sectional | Amhara | 27.6 | 71.2 | 28.8 | 44.2 | 55.8 | 86.6 | 13.4 | 260 | 26.7 | 12.4 | 46.7 | 14.2 | 53.3 | 39.1 | 7.6 | 83 | 17 | NR | NR | NR | NR | NR | NR | NR | YD and HT | August 15- September 24/2024 | | Yes |

Table 2: A table showing all data extracted from the primary research sources for the systematic review
