# Supplementary material for: Epidemiology of Traumatic brain injury in Ethiopia: A systematic review and meta-analysis of prevalence, mechanisms, and outcomes
Source: PLoS One. 2025 May 30;20(5):e0322641. doi: 10.1371/journal.pone.0322641 (PMC12124570; doi:10.1371/journal.pone.0322641)
Supplement: S3 Table — (DOCX) [file pone.0322641.s030.docx]

Table 3: The result of quality assessment of included studies using Joanna Briggs Institute (JBI)

| Author, Design, Year | Q1 | Q2 | Q3 | Q4 | Q5 | Q6 | Q7 | Q8 | Overall |
| --- | --- | --- | --- | --- | --- | --- | --- | --- | --- |
| Abebe et al, 2024 (23) | Yes | Yes | Yes | Yes | Yes | Yes | Yes | Yes | Yes |
| Aenderl et al, 2014 (34) | Yes | Yes | Yes | Yes | unclear | No | Yes | Yes | Yes |
| Amdeslasie et al, 2017 (24) | Yes | Yes | unclear | Yes | No | No | Yes | Yes | Yes |
| Assele et al, 2021 (25) | Yes | Yes | Yes | Yes | Yes | Yes | Yes | Yes | Yes |
| Ayele et al, 2024 (26) | Yes | Yes | Yes | Yes | Yes | Yes | Yes | Yes | Yes |
| Bedry et al, 2020 (27) | Yes | Yes | Yes | Yes | Yes | unclear | Yes | Yes | Yes |
| Biluts et al, 2017 (35) | Yes | Yes | Yes | Yes | Yes | unclear | Yes | Yes | Yes |
| Demlie et al, 2023 (28) | Yes | Yes | Yes | Yes | Yes | Yes | Yes | Yes | Yes |
| Dibera et al, 2024 (29) | Yes | Yes | Yes | Yes | Yes | Yes | Yes | Yes | Yes |
| Eshete et al, 2018(36) | Yes | Yes | Yes | Yes | Yes | Yes | Yes | Yes | Yes |
| Tesfaw et al, 2021(14) | Yes | Yes | Yes | Yes | Yes | Yes | Yes | Yes | Yes |
| Getabalew et al, 2023 (30) | Yes | Yes | Yes | Yes | Yes | Yes | Yes | Yes | Yes |
| Gezahegn et al, 2019 (37) | Yes | Yes | Yes | unclear | No | Yes | Yes | Yes | Yes |
| G/Michael et al, 2023 (31) | Yes | Yes | Yes | Yes | Yes | Yes | Yes | Yes | Yes |
| Hagos et al, 2022 (32) | Yes | Yes | Yes | Yes | Yes | Yes | Yes | Yes | Yes |
| Landes et al, 2017 (33) | Yes | Yes | Yes | Yes | unclear | unclear | Yes | Yes | Yes |
| Laeke et al, 2021(22) | Yes | Yes | Yes | Yes | unclear | unclear | Yes | Yes | Yes |
| Walle et al, 2016(38) | Yes | Yes | Yes | Yes | Yes | Yes | Yes | Yes | Yes |

Q1. Were the criteria for inclusion in the sample clearly defined?

Q2. Were the study subjects and the setting described in detail?

Q3. Was the exposure measured in a valid and reliable way?

Q4. Were objective, standard criteria used for measurement of the condition?

Q5. Were confounding factors identified?

Q6. Were strategies to deal with confounding factors stated?

Q7. Were the outcomes measured in a valid and reliable way?

Q8. Was appropriate statistical analysis used?
