# Supplementary material for: Epidemiology of Traumatic brain injury in Ethiopia: A systematic review and meta-analysis of prevalence, mechanisms, and outcomes
Source: PLoS One. 2025 May 30;20(5):e0322641. doi: 10.1371/journal.pone.0322641 (PMC12124570; doi:10.1371/journal.pone.0322641)
Supplement: S4 Table — (DOCX) [file pone.0322641.s031.docx]

| Outcome | Risk of Bias | Inconsistency | Indirectness | Imprecision | Publication Bias | Overall Certainty | Comments |
| --- | --- | --- | --- | --- | --- | --- | --- |
| Prevalence of TBI | Moderate | High | Low | Moderate | Suspected | Moderate | High I² suggests heterogeneity; limited regional representation |
| Causes of TBI (e.g., Assaults, RTIs, Falls) | Low | Moderate | Low | Low | Suspected | High | Clear patterns in causes, though minor heterogeneity |
| Severity (Mild, Moderate, Severe) | Low | Low | Low | Moderate | Low | High | Consistent GCS classification reduces inconsistency |
| Complications (e.g., seizures, infections) | Moderate | High | Low | Moderate | Suspected | Low | High heterogeneity and wide CIs; complications underreported |
| Surgical Intervention Rate | Moderate | Moderate | Low | Moderate | Low | Moderate | Rates are consistent but some regional variability observed |

**Table 4: Assessment of Certainty of Evidence Using the GRADE Approach**

Table 4: Assessment of Certainty of Evidence Using the GRADE Approach
