# Supplementary material for: Epidemiology of Traumatic brain injury in Ethiopia: A systematic review and meta-analysis of prevalence, mechanisms, and outcomes
Source: PLoS One. 2025 May 30;20(5):e0322641. doi: 10.1371/journal.pone.0322641 (PMC12124570; doi:10.1371/journal.pone.0322641)
Supplement: Attachment — Submitted filename: Detailed response to the reviewers.docx [file pone.0322641.s033.docx]

**Response to Reviewers and Editor**

We sincerely thank the reviewers and the editor for their thoughtful and constructive feedback on our manuscript, titled *“Traumatic Brain Injury in Ethiopia: A Systematic Review and Meta-Analysis.”* We greatly appreciate the time and effort invested in providing detailed comments, which have been invaluable in improving the quality and clarity of our work.

In this revised submission, we have carefully addressed all the comments raised by the reviewers and the editor in chief. Below, we provide a point-by-point response to each comment, outlining the revisions made and the rationale for these changes. We are confident that these revisions have strengthened the manuscript and aligned it more closely with the journal’s standards.

We look forward to your feedback and remain open to any further suggestions.

**To Academic editor Dr. Ukachukwu**

**Comment 1:**
"When submitting your revision, we need you to address these additional requirements. Please ensure that your manuscript meets PLOS ONE's style requirements, including those for file naming. The PLOS ONE style templates can be found at [link]."

**Response:**
Thank you for your guidance. We have tried to review the PLOS ONE style templates and ensured that our manuscript adheres to the journal's formatting requirements. We have tried to ensure that all elements of the manuscript align with the journal’s style and are ready for review.

**Comment 2:**
"Thank you for stating the following in your Competing Interests section: 'no'

Please complete your Competing Interests on the online submission form to state any Competing Interests. If you have no competing interests, please state 'The authors have declared that no competing interests exist.', as detailed online in our guide for authors."

**Response:**
Thank you for pointing this out. We confirm that the authors have no competing interests related to this study. Accordingly, we have updated the Competing Interests section in the manuscript to state:

*"The authors have declared that no competing interests exist."*

We have also included this statement in the cover letter for clarity. Please proceed with updating the online submission form on our behalf as indicated.

**Comment 3:**
"Please provide a complete Data Availability Statement in the submission form, ensuring you include all necessary access information or a reason for why you are unable to make your data freely accessible. If your research concerns only data provided within your submission, please write 'All data are in the manuscript and/or supporting information files' as your Data Availability Statement."

**Response:**
Thank you for your guidance. We have updated the Data Availability Statement to ensure compliance with PLOS ONE’s requirements. The statement now reads:

*"All data are included in the manuscript and/or supporting information files."*

If any additional clarifications are required regarding the Data Availability Statement, we are happy to provide further details.

**Comment 4:**
"Please include captions for your Supporting Information files at the end of your manuscript, and update any in-text citations to match accordingly. Please see our Supporting Information guidelines for more information."

**Response:**
Thank you for pointing this out. We have added captions for all Supporting Information files at the end of the manuscript, providing clear and concise descriptions for each file. Additionally, we have reviewed and updated all in-text citations to ensure they align with the Supporting Information guidelines provided by PLOS ONE. If any further adjustments or clarifications are required, we will be happy to make them**.**

**Comment 5:**

As required by our policy on Data Availability, please ensure your manuscript or supplementary information includes the following:

A numbered table of all studies identified in the literature search, including those that were excluded from the analyses.

For every excluded study, the table should list the reason(s) for exclusion.

**Response:**

Thank you for your insightful suggestion. We have added a detailed table summarizing the studies reviewed at the full-text screening stage, including the reasons for exclusion for studies not included in the final analysis. This table is now included in the supplementary file as **Table 1**.

Given the large number of studies initially identified (807 total), providing a table for every study, including those excluded at the title/abstract screening stage, would be impractical. Instead, we focused on the 41 studies assessed at the full-text review stage, as indicated in the PRISMA flowchart, and provided detailed reasons for excluding the 23 studies that did not meet our inclusion criteria.

We hope this approach aligns with your expectations, and we are happy to provide further details if needed.

**Comment 6:**
"If any of the included studies are unpublished, include a link (URL) to the primary source or detailed information about how the content can be accessed."

**Response:**
Thank you for your suggestion. None of the included studies in this systematic review and meta-analysis are unpublished. All studies were sourced from peer-reviewed journals and reputable databases, including PubMed, Scopus, Web of Science, and EMBASE. As such, no additional links or access information are required for unpublished studies.

**Comment 7:**
*A table of all data extracted from the primary research sources for the systematic review and/or meta-analysis. The table must include the following information for each study:

- Name of data extractors and date of data extraction
- Confirmation that the study was eligible to be included in the review
- All data extracted from each study for the reported systematic review and/or meta-analysis that would be needed to replicate your analyses.*

**Response:**
We thank the reviewer for highlighting this important point. In response, we have included a detailed table in our supplementary materials (see **Table 2**) that contains all the required information. Specifically:

1. **Name of data extractors and date of data extraction:**
   - This information is clearly documented in the columns titled "Data Extractors" and "Date of data extraction."
2. **Confirmation of eligibility for inclusion:**
   - The column "Article included in the study" explicitly indicates whether each study met the eligibility criteria for inclusion in the review.
3. **All extracted data needed to replicate analyses:**
   - The table comprehensively details all relevant data extracted from each study, including study characteristics (e.g., design, region, demographics), outcome variables (e.g., mortality rate, complications, length of hospital stay), and other relevant metrics used in the meta-analysis.

We tried to ensure transparency by providing all necessary information to replicate the analyses presented in the manuscript. We trust this addresses your concerns.

**Comment 8:**
If data or supporting information were obtained from another source (e.g., correspondence with the author of the original research article), please provide the source of data and dates on which the data/information were obtained by your research group.

**Response:**
We appreciate the reviewer’s request for clarity on the sources of our data. All data included in this systematic review and meta-analysis were extracted directly from the published articles identified through our systematic search process. No additional data or supporting information were obtained through correspondence with authors or other external sources.

**Comment 9:**
If applicable for your analysis, a table showing the completed risk of bias and quality/certainty assessments for each study or outcome. Please ensure this is provided for each domain or parameter assessed. For example, if you used the Cochrane risk-of-bias tool for randomized trials, provide answers to each of the signaling questions for each study. If you used GRADE to assess certainty of evidence, provide judgments about each of the quality-of-evidence factors. This should be provided for each outcome.

**Response:**
Thank you for the insightful comment. We have provided detailed tables outlining the risk of bias and quality/certainty assessments for the included studies.

1. **Risk of Bias Assessment:**
   - The risk of bias for each included study was evaluated using the Joanna Briggs Institute (JBI) critical appraisal checklist for observational studies.
   - Table 1 in the manuscript outlines the responses to each of the eight questions from the JBI checklist for all included studies.
   - The table provides a comprehensive assessment of risk across domains such as inclusion criteria, study population, measurement validity, and statistical methods.
2. **Certainty of Evidence Assessment:**
   - The certainty of evidence was assessed using the GRADE approach.
   - Table 3 in the manuscript summarizes the judgments for each GRADE domain (risk of bias, inconsistency, indirectness, imprecision, and publication bias) for the primary and secondary outcomes (e.g., prevalence, causes, complications, mortality).

These tables address all domains and parameters assessed, ensuring transparency in the evaluation of the included studies and outcomes.

Should further clarification or additional tables be required, we are happy to provide supplementary materials.

**Comment 10:**
An explanation of how missing data were handled. This information can be included in the main text, supplementary information, or relevant data repository. Please note that providing these underlying data is a requirement for publication in this journal, and if these data are not provided your manuscript might be rejected.

**Response:**
We appreciate the reviewer’s emphasis on the importance of transparency in handling missing data. In our systematic review and meta-analysis:

1. **Identification of Missing Data:**
   - During the data extraction process, some studies did not report specific variables (e.g., demographic details, certain outcomes such as complications or imaging findings). These instances were documented and flagged during data collection.
2. **Approach to Handling Missing Data:**
   - For studies where data on key outcomes were missing, these studies were excluded from the respective meta-analyses but were still considered in qualitative synthesis if they met the inclusion criteria.
   - Missing data were not imputed to ensure the validity of pooled estimates and to avoid introducing bias.
3. **Documentation of Missing Data:**
   - Tables summarizing the characteristics and results of included studies (e.g., Table 2 on the supplementary file ) explicitly indicate where data were not reported (e.g., marked as "NR" for "Not Reported").
   - A detailed description of how missing data influenced the analysis is provided in the "Limitations" section of the Discussion.
4. **Data Availability:**
   - All underlying data and extracted variables are provided in the supplementary materials to ensure full transparency and compliance with journal requirements.

If additional details or clarifications are needed, we will gladly include further explanations in the supplementary information or provide raw data via a relevant repository.

**Comment 11:**
PLOS authors have the option to publish the peer review history of their article (what does this mean?). If published, this will include your full peer review and any attached files. If you choose “no,” your identity will remain anonymous but your review may still be made public.

**Response:**
Thank you for providing this option. We choose to publish the peer review history of our article. We believe that sharing the peer review process will enhance the transparency and credibility of our work and contribute to the open dissemination of scientific knowledge.

**Response to Dr. Adel Elnashar**

**Comment 1**:

Please include more info regarding your strategy to collect the demographics and do the meta-analysis specifically in Ethiopia, which will be useful to anyone who is s a foreigner to the policies and census in Ethiopia. There was no details regarding how to you were able confirm these numbers. Resources and reference for confirmation would have been very helpful.

**Response to Reviewer #1:**

Thank you for your valuable feedback. We appreciate your suggestion to include more information regarding the strategy for collecting demographic data and conducting the meta-analysis, particularly for readers unfamiliar with Ethiopian policies and census data. Below, we have incorporated additional details into the methodology section of the manuscript. We have also provided references and clarified how the numbers were verified to enhance transparency and reproducibility.

**Comment 2:**

I understand you included references "at times", but why do you pick these studies, would have been a very helpful input

**Response:**

Thank you for your feedback. In addition to the database search, we also conducted a manual review of the reference lists of pertinent articles and reviews. This approach ensured the inclusion of studies that might not have been indexed in the primary databases. We have now elaborated on this process in the methodology section to provide more clarity.

**Comment 3:**
"Rewrite the demographics section. For example, stating that males are 78% and females are 22% is redundant. A more useful piece of info is how these populations in the different studies differed in every study when compared to the whole and why."

**Response:**
Thank you for your valuable feedback. We have rewritten the demographics section to provide a more meaningful analysis of the data. The revised section now highlights the gender and residence distribution across the included studies, emphasizing patterns and differences in rural versus urban settings and their association with TBI causes. This approach removes redundancy and adds interpretive context, aligning the demographic findings with the overarching themes of the manuscript. We hope the revised section addresses your concerns effectively. Please let us know if additional adjustments are required.

**Comment 4:**
"Please summarize the confidence intervals. I prefer this info to be in a table, for comparison. At times, it was wordy and not helpful."

**Response:**
Thank you for your feedback. We have addressed your suggestion by summarizing the key findings and their confidence intervals in a concise table format. This table facilitates direct comparison and eliminates redundancy, enhancing clarity and readability. The updated table has been incorporated into the results section (Table 2).

**Comment 5:**

Better describe figures. e.g. Figure 2: Forest plot of assault/violence as a cause of TBI in Ethiopia this should have been Figure 2: assault/violence as a cause of TBI in Ethiopia. Above is a Forest plot describing so and so.

**Response:**

Thank you for your insightful comment regarding the figure descriptions. We have revised all figure captions to provide more detailed explanations of their content and significance. These revised captions now describe the context, the type of data presented (e.g., pooled estimates and confidence intervals), and any notable observations, such as heterogeneity among studies. We believe these changes enhance the clarity and accessibility of the figures for the readers.

**Comment 6:**

I would suggest changing the name of the study e.g. "the prevalence of TBI in Ethiopia" or " comparing TBI prevalence in Ethiopia to the rest of sub-Saharan Africa", "The mechanism of TBI in Ethiopia" or whatever the authors prefer.

**Response:**

Thank you for your thoughtful suggestion regarding the title of the study. We agree that the title can be improved to better reflect the scope and focus of our review. In response, we have revised the title to **"Epidemiology of Traumatic Brain Injury in Ethiopia: A Systematic Review and Meta-Analysis of Prevalence, Mechanisms, and Outcomes."** This updated title highlights the key aspects of the study, ensuring clarity and alignment with the objectives of the review. We hope this change meets your expectations.

**Comment 7:**

There was no in depth analysis for the data in the supplementary material when it relates in Ethiopia, but only a referral to why the numbers are low in other countries per their reference.

**Response:**

Thank you for your valuable feedback. We appreciate your observation regarding the analysis of the data in the supplementary material. To address this, we have now included a more in-depth analysis of the data specific to Ethiopia including subgroup analysis. This analysis highlights the unique factors contributing to the observed trends and provides a comprehensive understanding of the local context. We hope this addition meets your expectations and enhances the overall quality of our work.

**Comment 8:**
The JBI approach and GRADE tables were great efforts, and referring to them is helpful but should have better suited in the supplementary material, as they are not the focus of this study, and rather bring more info from the supplementary to the main other than the mere causes and mortality rate. Still, it can be included in a paragraph or two as a summary.

**Response:**
Thank you for acknowledging our efforts in utilizing the JBI approach and GRADE tables. We agree that their detailed presentation is more suited to the supplementary material. As per your suggestion, we have relocated the full tables to the supplementary section and included a concise summary in the main manuscript. This summary highlights the key findings related to study quality and evidence certainty, ensuring the focus remains on the primary outcomes of the study.

**Response to Reviewer 2:**

First of all, we would like to appreciate your recognition of the rigorous methodology employed in this study and the efforts made to ensure a comprehensive data collection process. Your acknowledgment of the use of PRISMA guidelines, the JBI checklist, the GRADE approach, and statistical rigor through Stata 17 motivates us to continue adhering to the highest standards of systematic review and meta-analysis.

# Comment 1:

The studies included in the review are disproportionately concentrated in certain regions, such as Addis Ababa. This uneven distribution may lead to a biased estimation of the national TBI burden, as rural and underserved areas might have different TBI patterns and risk factors.

**Response:**
Thank you for highlighting this important limitation. We agree that the uneven regional distribution of studies poses a challenge to achieving a fully representative national estimate of TBI burden in Ethiopia. This issue has been acknowledged in the limitations section of our manuscript, where we have discussed the lack of data from rural and underserved areas and its potential impact on the findings.

To address this concern more explicitly, we have emphasized the need for future research to focus on large-scale, nationally representative studies. Additionally, we have included the following recommendations in the discussion section:

- “Encourage stratified sampling techniques to ensure proportional representation of regions with varying levels of healthcare access.
- Promote collaboration with local healthcare providers and community organizations to facilitate data collection in underserved and rural areas.”

We hope these adjustments and explicit recommendations address your concern adequately.

**Comment 2:**
"By relying solely on published studies, the meta-analysis is susceptible to publication bias. Studies with significant results are more likely to be published, potentially skewing the overall picture. To mitigate this, the authors could consider exploring unpublished studies, such as those presented in conference proceedings or registered in trial databases. Grey literature searches can also be incorporated to capture a more complete range of research findings. This would provide a more balanced and accurate assessment of the TBI situation in Ethiopia."

**Response:**
Thank you for raising this concern about publication bias. We acknowledge that relying on published studies may introduce bias, as studies with significant findings are more likely to be published. To address this, we have incorporated funnel plots and Egger’s test to assess publication bias, as noted in the manuscript. While these tools provide insights into the potential presence of bias, we agree that including unpublished studies and grey literature could improve the comprehensiveness of our review.

Although unpublished studies and grey literature searches were beyond the scope of the current analysis, we have emphasized this as a recommendation for future research in the discussion section. Specifically, we propose the inclusion of:

- Conference proceedings.
- Registered trial databases such as clinicaltrials.gov.
- Grey literature sources, such as theses or institutional reports, which may provide valuable insights from regions currently underrepresented in published research.

These additions would enhance the breadth and balance of evidence, ensuring a more accurate assessment of the TBI burden in Ethiopia.

**Comment 3:**
"Many of the included studies lack access to advanced imaging modalities like CT or MRI, particularly in rural settings. This deficiency may result in the underreporting of TBI severity and complexity, as milder cases or subtle brain injuries could go undetected. Future investigations should aim to improve diagnostic capabilities in these areas. This could involve providing training and resources for healthcare workers to better identify and document TBI cases. Mobile diagnostic units equipped with appropriate imaging technology could also be deployed to reach rural and remote areas, ensuring more accurate diagnosis and a more comprehensive understanding of the true prevalence and nature of TBI."

**Response:**
Thank you for highlighting this critical issue. We agree that the lack of access to advanced imaging modalities, especially in rural settings, poses significant limitations in diagnosing and accurately reporting TBI cases. This limitation is acknowledged in the manuscript, particularly in the discussion and limitations sections, where we address the potential underreporting of milder or subtle brain injuries. To align with your suggestions, we have expanded the discussion to include the following recommendations:

- **Capacity Building:** Providing training and resources for healthcare workers in rural and underserved areas to enhance their ability to identify and document TBI cases using available tools.
- **Mobile Diagnostic Units:** Advocating for the implementation of mobile diagnostic units equipped with portable imaging technologies, such as portable CT or point-of-care ultrasound, to improve diagnostic coverage in remote areas.
- **Resource Allocation:** Encouraging policymakers and stakeholders to prioritize the allocation of resources to expand access to advanced imaging modalities in regional and rural hospitals.

We hope these additions address your concern and reinforce the importance of addressing diagnostic limitations in future research and healthcare strategies.

**Comment 4:**
"The variability in trauma care availability across Ethiopia is not adequately accounted for in the current analysis. Different levels of healthcare infrastructure and resources can significantly impact patient outcomes, including mortality and complication rates. To rectify this, future studies should incorporate trauma care availability as a variable in the analysis. This could involve collecting data on factors such as the number of trained healthcare providers, availability of critical care facilities, and access to emergency medical services in different regions. By adjusting for these factors, a more accurate assessment of the impact of TBI and the effectiveness of interventions can be achieved.”

**Response:**
Thank you for this insightful observation. We acknowledge that variability in trauma care availability across Ethiopia likely influences patient outcomes, including mortality and complication rates, and was not explicitly analyzed in our current study. In the limitations section, we have highlighted the disparity in healthcare infrastructure as a potential confounder that could impact our findings. To address this concern more comprehensively, we have added the following points to the discussion section:

- Emphasizing the need for future studies to collect and incorporate data on trauma care availability, such as the number of trained healthcare providers, the presence of critical care facilities, and the accessibility of emergency medical services.
- Proposing that future research stratify analyses based on regional healthcare resources to account for disparities in trauma care availability.
- Recommending collaborations with regional health authorities to better understand and quantify the influence of trauma care variability on TBI outcomes.

**Response to Dr. Syed M. Faisal**

**Comment 1:**

Authors acknowledge the data heterogeneity (e.g., I² values), they should explore subgroup analyses based on geographical regions, study design, or study population demographics to provide insights into the variability.

**Response:**

Thank you for your valuable feedback. We have thoroughly addressed the issue of data heterogeneity (e.g., I² values) by conducting detailed subgroup analyses to explore the variability based on geographical regions, study design, and demographic factors, as suggested.

1. **Geographical Regions:**
   We performed a region-based subgroup analysis and highlighted significant variations in the causes of TBI across Ethiopia. For example, road traffic accidents were identified as a major contributor in all regions, with Sidama and Amhara regions showing notably higher effect sizes. Similarly, falls and interpersonal violence were prominent causes in Amhara, while occupational hazards were particularly significant in Addis Ababa. These findings underscore the importance of region-specific public health interventions.
2. **Study Design:**
   We examined subgroup differences based on study design and found substantial heterogeneity in retrospective (I² = 96.13%) and cross-sectional studies (I² = 97.96%), likely due to differences in methodologies and populations. In contrast, prospective studies demonstrated no heterogeneity (I² = 0.00%), indicating greater consistency in this subgroup. This highlights the importance of methodological considerations when interpreting pooled results.
3. **Study Population Demographics:**
   While sex and place of residence did not show statistically significant subgroup differences, we observed consistently high heterogeneity (I² values) within these subgroups. This suggests that additional, unexplored factors beyond demographics likely contribute to the variability in TBI causes, warranting further research.

In summary, we have acknowledged and explored the sources of heterogeneity through subgroup analyses, providing meaningful insights into the variability. These findings are now emphasized in the manuscript to address your comments thoroughly.

**Comment 2:**

Include a funnel plot or Egger’s test results in the manuscript to substantiate the claim of publication bias assessment.

**Response:**

We appreciate your suggestion to include a funnel plot or Egger’s test results to substantiate the publication bias assessment.

In response, we have incorporated a Egger’s regression test to evaluate the potential for publication bias in our analysis of the prevalence and causes of traumatic brain injury (TBI). Egger’s regression test results for the causes of TBI (road traffic accidents, falls, and occupational hazards) indicate no statistically significant publication bias. These findings suggest that while there is evidence of publication bias in the prevalence data, the observed effects related to the causes of TBI are unlikely to be influenced by selective reporting of positive results. We have included these findings in the revised manuscript to enhance the transparency and rigor of our publication bias assessment. Thank you for your valuable feedback.

**Comment 3:**

Clarify how mortality rates and complications were standardized across studies. Were these definitions uniform across studies, or did variability exist?

**Response:**

Thank you for raising this important point. The definitions of mortality rates and complications varied across the included studies. While some studies provided consistent definitions, others exhibited variability in the criteria used. **Mortality Rate:** It was defined as the proportion of patients who died due to traumatic brain injury (TBI) within the timeframe reported by each individual study. This timeframe varies across studies, potentially introducing heterogeneity.

**Complication Definition:** Complications included any adverse events or conditions reported by the included studies that arose after the initial TBI. This broad definition encompasses a wide range of potential complications, and the specific conditions considered may not be uniform across all studies.

**Standardization Limitations:** Due to variations in reporting methods and the availability of detailed information across studies, complete standardization of mortality rates and complication data was not feasible. **While every effort was made to harmonize data and ensure consistency, some degree of heterogeneity likely remains.**

Future research endeavors should prioritize standardized definitions and reporting guidelines for mortality rates and complications in TBI studies. This will enable more robust and accurate comparisons and meta-analyses in the future, contributing to a more comprehensive understanding of TBI outcomes.

**Comment 4:**
"The lack of representation from certain Ethiopian regions, particularly rural and underserved areas, is a limitation. Highlight how this could impact the generalizability of findings."

**Response:**
Thank you for this valuable comment. We agree that the lack of representation from certain Ethiopian regions, particularly rural and underserved areas, is a notable limitation of our study. This limitation may impact the generalizability of the findings, as rural populations often have unique risk factors, healthcare access challenges, and socioeconomic conditions that could influence the prevalence, causes, and outcomes of traumatic brain injury. We have now added this point under the "Limitations" section of the manuscript to provide a more comprehensive discussion of the potential implications.

**Comment 5:**
"Expand on the implications for Ethiopian health policy and trauma care systems. For example, how might these findings inform resource allocation or prevention strategies for road traffic accidents and violence?"

**Response:**
Thank you for your insightful suggestion. We have expanded the "Implications" section of the manuscript to discuss how our findings can inform Ethiopian health policy and trauma care systems. Specifically, we highlight the potential for these findings to guide resource allocation and the development of targeted prevention strategies, particularly for road traffic accidents and violence, which were identified as significant contributors to traumatic brain injury. We have also emphasized the importance of strengthening trauma care infrastructure, improving pre-hospital care, and implementing public health campaigns to raise awareness. This addition provides a more comprehensive understanding of the policy relevance of our findings.

**Comment 6:**
"Rephrase ambiguous sentences for clarity, e.g., '... insufficient healthcare infrastructure... exacerbated by limited access to specialized medical services' could be refined for readability."

**Response:**
Thank you for pointing this out. We have carefully reviewed the manuscript and revised ambiguous sentences to improve clarity and readability. For instance:

- The phrase “... insufficient healthcare infrastructure... exacerbated by limited access to specialized medical services” has been revised to:
  “... compounded by inadequate healthcare infrastructure, restricted access to specialized medical services, and limited resources.”

**Comment 7:**

"Include exact sample sizes (n) for all pooled estimates in tables and figure legends."

**Response:**
Thank you for your valuable comment. We have now included the exact sample sizes (n) for all pooled estimates in the legends of the respective tables and figures. Specifically, for each pooled estimate (e.g., road traffic accidents, falls, interpersonal violence, and occupational hazards), the total sample size across the studies included in the meta-analysis has been calculated and reported. For example:

- In Table 3, we now provide pooled proportions along with the total sample size (n) for each cause of traumatic brain injury (TBI).
- In Figures, the forest plot legend has been updated to specify the total sample size (n) for the pooled estimates presented.

These additions ensure clarity and transparency regarding the data used in our analysis. Please refer to the revised manuscript for these updates.

**Comment 8:**
"Verify the inclusion of all mandatory PRISMA elements, particularly in the supplementary material."

**Response:**
Thank you for your observation. We have cross-checked our manuscript and supplementary material against the PRISMA checklist to ensure that all mandatory elements are included.

**Comment 9:**
"Provide additional details on statistical corrections for heterogeneity, if any were used."

**Response:**
Thank you for raising this important point. To address heterogeneity, we employed the following statistical methods:

1. **Random-Effects Model:**
   - A random-effects model was used to account for the variability across studies, assuming that differences in study populations, methodologies, and settings contribute to heterogeneity. This approach provides more conservative pooled estimates by considering both within-study and between-study variation.
2. **Subgroup Analyses:**
   - To explore the sources of heterogeneity, we performed subgroup analyses based on study design (e.g., retrospective, cross-sectional, prospective), geographical region, and patient demographics (e.g., age, gender). These analyses helped identify patterns and potential drivers of heterogeneity.
3. **I² and Cochran’s Q Tests:**
   - We quantified heterogeneity using the I² statistic, with thresholds for low, moderate, and high heterogeneity, and Cochran’s Q test, assessing whether observed variability was greater than expected by chance.
4. **Publication Bias Assessment:**
   - Funnel plots and Egger’s regression test were used to detect potential publication bias, which can contribute to heterogeneity.

These details have been added to the "Statistical Analysis" section of the Methods to clarify our approach to handling heterogeneity.

**Comment 10:**

Ensure recent references are cited for comparisons with other LMICs or African nations.

**Response:**

Thank you for this insightful suggestion. We have reviewed and updated the manuscript to include more recent references (primarily from the past five years) to strengthen the comparisons with other low- and middle-income countries (LMICs) and African nations. These updated references have been incorporated into sections [mention the specific sections or subsections] to provide a more current and comprehensive context for our analysis. We appreciate the opportunity to enhance the relevance and rigor of the manuscript with these updates.

Finally, we sincerely hope that the revisions and responses provided address the reviewers' and editor’s concerns effectively. However, we remain fully committed to ensuring the highest quality of our manuscript. If further revisions or clarifications are needed, we are more than willing to re-revise the manuscript as necessary. We greatly appreciate your continued guidance and support throughout this process and look forward to your feedback.
