# Supplementary material for: Epidemiology of Traumatic brain injury in Ethiopia: A systematic review and meta-analysis of prevalence, mechanisms, and outcomes
Source: PLoS One. 2025 May 30;20(5):e0322641. doi: 10.1371/journal.pone.0322641 (PMC12124570; doi:10.1371/journal.pone.0322641)
Supplement: Attachment — Submitted filename: Detailed_response_to_the_reviewers_auresp_2.docx [file pone.0322641.s034.docx]

**Response to Reviewers and Editor**

We sincerely thank the reviewers and the editor for their thoughtful and constructive feedback on our manuscript, titled “**Epidemiology of Traumatic Brain Injury in Ethiopia: A Systematic Review and Meta-Analysis of Prevalence, Mechanisms, and Outcomes**.” We greatly appreciate the time and effort invested in providing detailed comments, which have been invaluable in improving the quality and clarity of our work.

In this revised submission, we have carefully addressed all the comments raised by the reviewer. Below, we provide a point-by-point response to each comment, outlining the revisions made and the rationale for these changes.

We look forward to your feedback and remain open to any further suggestions.

**Response to Dr. Adel Elnashar**

**Comment 1**:
"Remove all forest plots and in a simple table list all the heterogeneity results for the different variables in question. The plot shows the heterogeneity, but in themselves don’t add much information, can be included in supplementary."

**Response**:
In response to your comment, we have removed all forest plots from the main text and replaced them with a comprehensive summary table that lists the pooled estimates, confidence intervals, heterogeneity (I²), and p-values for all key variables analyzed in the study (Table 3). This table provides a clear and concise overview of the heterogeneity results, making the findings more accessible to readers.

Additionally, all forest plots have been moved to the Supplementary Materials section, where they are labeled and referenced appropriately (Supplementary Figures 1–31). This change streamlines the main manuscript while ensuring that the detailed graphical representations remain available for readers who wish to explore the data further.

**Comment 2**:
"In the introduction, there is a consistent jump back and forth to the prevalence of TBI and the impact of TBI."

**Response**:
In response to your comment, we have thoroughly revised the Introduction to ensure a logical and coherent flow, avoiding unnecessary jumps between the prevalence and impact of TBI.

**Comment 3**:
"The variable inconsistency between retrospective and prospective studies doesn’t indicate that what you mentioned. These variables are indeed expected to produce this effect, but your mentioned conclusion can’t be deducted from this input, unless the prospective studies shared population, methodology, etc. Is this the case? Include then."

**Response**:
In response to your comment, we have revised the manuscript to provide a more nuanced interpretation of the heterogeneity between retrospective and prospective studies. Specifically, we have clarified that the lack of heterogeneity in prospective studies may be influenced by factors such as smaller sample sizes, similarities in population characteristics, and standardized data collection protocols, rather than inherent consistency in study design. For example, the prospective studies included in our analysis were conducted in similar urban settings and utilized comparable methodologies, which may have contributed to the observed consistency. We have also acknowledged that further research is needed to confirm these findings and explore additional factors that may influence heterogeneity. (see pages 7 (lines 240-247) and ).

**Comment 4**:
"Having your own mortality rate definition is confusing, provide a different term."

**Response**:
In response to your comment, we have revised the manuscript to use a more standard term for mortality rate. Specifically, we have replaced the custom definition with **"**short-term mortality rate**"**, which we define as the proportion of patients who died due to traumatic brain injury (TBI) within the timeframe reported by each individual study (e.g., in-hospital mortality or 30-day mortality). This change improves clarity and ensures consistency with standard terminology in the field. The revised definition has been incorporated into the Methods (page 5, lines 163-166). Thank you for this insightful suggestion, which has enhanced the readability of the manuscript.

**Comment 5**:
"Your study has significant inconsistency, I2, this should your number one limitation, and the first recommendations should address this subject. Your discussion should also include it in your first to second paragraph as it reflects on all your input and conclusions."

**Response**:
In response to your comment, we have revised the manuscript to highlight **heterogeneity (I²)** as the primary limitation and address it early in the **Discussion** and **Recommendations** sections. Specifically, we have:

1. Moved the discussion of heterogeneity to the **top of the Limitations section**, emphasizing its impact on the generalizability and reliability of the findings (Page 17, lines 519-525).
2. Added a detailed discussion of heterogeneity in the **first paragraph of the Discussion**, including potential sources (e.g., regional variations, study design differences) and their implications for the interpretation of the results (Page 13, lines 353-358).
3. Made the **first recommendation** about addressing heterogeneity in future studies, suggesting strategies such as standardized data collection methods and stratified analyses (Page 17, lines 535-538).

**Comment 6**:
"The paragraph with the heterogeneity and the publication bias, include each separately, they are unrelated, only include the numbers under results, and include your suggestions and reflections under discussions."

**Response**:
We sincerely thank the reviewer for this valuable suggestion. In response to your comment, we have separated the discussion of **heterogeneity** and **publication bias** into distinct sections. Specifically, we have:

1. Included only the numerical results (e.g., I² values, Egger’s test p-values) in the **Results** section, presenting them in a clear and concise manner.
2. Moved the interpretation and reflections on heterogeneity and publication bias to the **Discussion** section, where we discuss their implications for the findings and recommendations for future research. (page 8/9, lines 303-313, Page 13, lines 353-363)

**Comment 7**:
"Egger test coding can also be included under supplementary."

**Response**:
We sincerely thank the reviewer for this suggestion. In response to your comment, we have moved the Egger’s test figures (e.g., funnel plots) to the Supplementary Materials

**Comment 8**:
"Surgical intervention doesn’t reflect severity, indeed many of the most severe TBI aren’t warranted for surgery. Surgery is a tool used when it is indicated. A slight midline shift may indicate a surgery need, but a more severe bleed, may not benefit from surgery."

**Response**:
We sincerely thank the reviewer for this important observation. In response to your comment, we have revised the manuscript to clarify that **surgical intervention rates** are not a direct measure of TBI severity but instead reflect the proportion of cases where surgery was clinically indicated (Page 7, 230-235, and Page 15, lines 467-475). Specifically, we have:

1. Emphasized that surgical intervention is based on specific clinical indications (e.g., epidural or subdural hematomas, midline shift, elevated intracranial pressure) rather than injury severity alone.
2. Discussed the variability in surgical intervention rates across studies, which may be influenced by differences in clinical practices, resource availability, and patient populations.
3. Provided context for the common indications for surgery in TBI and how these relate to the findings of our study.

**Comment 9**:
"Don’t use GCS to classify TBI severity in your study unless isolated, GCS can vary depending on general state, or otherwise list GCS ranges without referring to TBI severity."

**Author Response**:
In response to your comment, we have revised the manuscript to avoid using the Glasgow Coma Scale (GCS) to classify TBI severity

**Comment 10:**
There were multiple outcome variables added in your new manuscript. There were multiple versions, but after adding this, there was no consistency in using its elements for analysis. Please review these outcomes or remove."

**Response:**

We sincerely appreciate the reviewer’s careful assessment of our manuscript and their constructive feedback. We acknowledge the concern regarding multiple outcome variables and the need for consistency in their application throughout the analysis (page 5-8).

To address this, we have made the following modifications:

1. **Clearly Defined Outcome Measures**:
   - We have categorized primary and secondary outcomes to ensure clarity and coherence.
   - The primary outcomes include the pooled prevalence of TBI, leading causes (assaults, RTIs, falls), and overall mortality rate.
   - The secondary outcomes now explicitly include GCS classification**,** complications (seizures, infections, aspiration pneumonia, wound infections), surgical intervention rate, imaging findings, and regional/demographic variations in TBI prevalence and outcomes**.**
2. **Standardized Definitions for Key Outcomes**:
   - Short-term mortality is now clearly defined as the proportion of TBI-related deaths reported within each study’s specific follow-up period (e.g., in-hospital mortality or 30-day mortality). This standardization ensures comparability.
   - Complications are now explicitly categorized based on available data, preventing inconsistencies in reporting.
3. **Consistency in Subgroup Analyses**:
   - The subgroup analyses are now fully aligned with the defined outcome measures, ensuring coherence across different analyses.
   - The surgical intervention rate is analyzed separately from TBI severity, clarifying that it reflects cases requiring neurosurgical procedures rather than serving as a direct severity indicator.
4. **Addressing Heterogeneity and Variability**:
   - We have acknowledged and discussed the variability in study designs (retrospective, prospective, and cross-sectional), explaining how differences in methodology, population characteristics, and healthcare access contribute to heterogeneity.
   - Regional differences are explored systematically, particularly in relation to mortality, complication rates, and surgical intervention rates, ensuring that the reported outcomes remain interpretable.

By implementing these revisions, we have improved the **clarity, structure, and consistency** of the outcome measures throughout the manuscript. We believe these changes effectively address the reviewer’s concern and enhance the rigor of our analysis.

We appreciate your valuable insights and welcome any further feedback to strengthen the manuscript.
