# Supplementary material for: Epidemiology of Traumatic brain injury in Ethiopia: A systematic review and meta-analysis of prevalence, mechanisms, and outcomes
Source: PLoS One. 2025 May 30;20(5):e0322641. doi: 10.1371/journal.pone.0322641 (PMC12124570; doi:10.1371/journal.pone.0322641)
Supplement: Peer review history

# Author comment: Author response to Decision Letter 0

**DOI:** 10.1371/journal.pone.0322641.r001

8 Nov 2024

# Peer review: Decision Letter 0

**DOI:** 10.1371/journal.pone.0322641.r002

**Authors:** Alvan-Emeka K. Ukachukwu

30 Dec 2024

PONE-D-24-50934Traumatic Brain Injury in Ethiopia, A Systematic Review and Meta-analysisPLOS ONE

Dear Dr. Molla,

Thank you for submitting your manuscript to PLOS ONE. After careful consideration, we feel that it has merit but does not fully meet PLOS ONE’s publication criteria as it currently stands. Therefore, we invite you to submit a revised version of the manuscript that addresses the points raised during the review process.

**See reviewer comments below.**

Please submit your revised manuscript by Feb 13 2025 11:59PM. If you will need more time than this to complete your revisions, please reply to this message or contact the journal office at plosone@plos.org . When you're ready to submit your revision, log on to https://www.editorialmanager.com/pone/ and select the 'Submissions Needing Revision' folder to locate your manuscript file.

Please include the following items when submitting your revised manuscript:

- A rebuttal letter that responds to each point raised by the academic editor and reviewer(s). You should upload this letter as a separate file labeled 'Response to Reviewers'.
- A marked-up copy of your manuscript that highlights changes made to the original version. You should upload this as a separate file labeled 'Revised Manuscript with Track Changes'.
- An unmarked version of your revised paper without tracked changes. You should upload this as a separate file labeled 'Manuscript'.

If you would like to make changes to your financial disclosure, please include your updated statement in your cover letter. Guidelines for resubmitting your figure files are available below the reviewer comments at the end of this letter.

If applicable, we recommend that you deposit your laboratory protocols in protocols.io to enhance the reproducibility of your results. Protocols.io assigns your protocol its own identifier (DOI) so that it can be cited independently in the future. For instructions see: https://journals.plos.org/plosone/s/submission-guidelines#loc-laboratory-protocols . Additionally, PLOS ONE offers an option for publishing peer-reviewed Lab Protocol articles, which describe protocols hosted on protocols.io. Read more information on sharing protocols at https://plos.org/protocols?utm_medium=editorial-email&utm_source=authorletters&utm_campaign=protocols .

We look forward to receiving your revised manuscript.

Kind regards,

Alvan-Emeka K. Ukachukwu, MD, MSc.GH

Academic Editor

PLOS ONE

**Journal Requirements:**

1. When submitting your revision, we need you to address these additional requirements.

Please ensure that your manuscript meets PLOS ONE's style requirements, including those for file naming. The PLOS ONE style templates can be found at 

https://journals.plos.org/plosone/s/file?id=wjVg/PLOSOne_formatting_sample_main_body.pdf and 

https://journals.plos.org/plosone/s/file?id=ba62/PLOSOne_formatting_sample_title_authors_affiliations.pdf

2. Thank you for stating the following in your Competing Interests section:  

"no"

Please complete your Competing Interests on the online submission form to state any Competing Interests. If you have no competing interests, please state "The authors have declared that no competing interests exist.", as detailed online in our guide for authors at http://journals.plos.org/plosone/s/submit-now 

 This information should be included in your cover letter; we will change the online submission form on your behalf.

3. Please provide a complete Data Availability Statement in the submission form, ensuring you include all necessary access information or a reason for why you are unable to make your data freely accessible. If your research concerns only data provided within your submission, please write "All data are in the manuscript and/or supporting information files" as your Data Availability Statement.

4. Please include captions for your Supporting Information files at the end of your manuscript, and update any in-text citations to match accordingly. Please see our Supporting Information guidelines for more information: http://journals.plos.org/plosone/s/supporting-information. 

5. As required by our policy on Data Availability, please ensure your manuscript or supplementary information includes the following: 

A numbered table of all studies identified in the literature search, including those that were excluded from the analyses.  

For every excluded study, the table should list the reason(s) for exclusion.  

If any of the included studies are unpublished, include a link (URL) to the primary source or detailed information about how the content can be accessed. 

A table of all data extracted from the primary research sources for the systematic review and/or meta-analysis. The table must include the following information for each study: 

Name of data extractors and date of data extraction 

Confirmation that the study was eligible to be included in the review.  

All data extracted from each study for the reported systematic review and/or meta-analysis that would be needed to replicate your analyses. 

If data or supporting information were obtained from another source (e.g. correspondence with the author of the original research article), please provide the source of data and dates on which the data/information were obtained by your research group. 

If applicable for your analysis, a table showing the completed risk of bias and quality/certainty assessments for each study or outcome.  Please ensure this is provided for each domain or parameter assessed. For example, if you used the Cochrane risk-of-bias tool for randomized trials, provide answers to each of the signalling questions for each study. If you used GRADE to assess certainty of evidence, provide judgements about each of the quality of evidence factor. This should be provided for each outcome.  

An explanation of how missing data were handled. 

This information can be included in the main text, supplementary information, or relevant data repository. Please note that providing these underlying data is a requirement for publication in this journal, and if these data are not provided your manuscript might be rejected.  

**Additional Editor Comments:**

The manuscript requires significant revisions as noted by the reviewers. Kindly provide detailed responses to the reviewer comments.

[Note: HTML markup is below. Please do not edit.]

Reviewers' comments:

Reviewer's Responses to Questions

**Comments to the Author**

1. Is the manuscript technically sound, and do the data support the conclusions?

The manuscript must describe a technically sound piece of scientific research with data that supports the conclusions. Experiments must have been conducted rigorously, with appropriate controls, replication, and sample sizes. The conclusions must be drawn appropriately based on the data presented. 

Reviewer #1: Yes

Reviewer #2: Yes

Reviewer #3: Yes

**********

2. Has the statistical analysis been performed appropriately and rigorously? 

Reviewer #1: Yes

Reviewer #2: Yes

Reviewer #3: Yes

**********

3. Have the authors made all data underlying the findings in their manuscript fully available?

The PLOS Data policy requires authors to make all data underlying the findings described in their manuscript fully available without restriction, with rare exception (please refer to the Data Availability Statement in the manuscript PDF file). The data should be provided as part of the manuscript or its supporting information, or deposited to a public repository. For example, in addition to summary statistics, the data points behind means, medians and variance measures should be available. If there are restrictions on publicly sharing data—e.g. participant privacy or use of data from a third party—those must be specified.

Reviewer #1: Yes

Reviewer #2: Yes

Reviewer #3: Yes

**********

4. Is the manuscript presented in an intelligible fashion and written in standard English?

PLOS ONE does not copyedit accepted manuscripts, so the language in submitted articles must be clear, correct, and unambiguous. Any typographical or grammatical errors should be corrected at revision, so please note any specific errors here.

Reviewer #1: Yes

Reviewer #2: Yes

Reviewer #3: Yes

**********

5. Review Comments to the Author

Please use the space provided to explain your answers to the questions above. You may also include additional comments for the author, including concerns about dual publication, research ethics, or publication ethics. (Please upload your review as an attachment if it exceeds 20,000 characters)

**Reviewer #1:**  1. Please include more info regarding your strategy to collect the demographics and do the meta-analysis specifically in Ethiopia, which will be useful to anyone who is s a foreigner to the policies and census in Ethiopia. There was no details regarding how to you were able confirm these numbers. Resources and reference for confirmation would have been very helpful. I understand you included references "at times", but why do you pick these studies, would have been a very helpful input.

2. Rewrite the demographics section. For example, stating that males are 78, females are 22% is redundant. A more useful piece of info is how these populations in the different studies differed in every study when compared to the whole and why.

3. Please summarize the confidence intervals. I prefer this info to be in a table, for comparison. At times, it was wordy and not helpful.

4. Better describe figures. e.g. Figure 2: Forest plot of assault/violence as a cause of TBI in Ethiopia this should have been Figure 2: assault/violence as a cause of TBI in Ethiopia. Above is a Forest plot describing so and so.

5. I would suggest changing the name of the study e.g. "the prevalence of TBI in Ethiopia" or " comparing TBI prevalence in Ethiopia to the rest of sub-Saharan Africa", "The mechanism of TBI in Ethiopia" or whatever the authors prefer.

6. There was no in depth analysis for the data in the supplementary material when it relates in Ethiopia, but only a referral to why the numbers are low in other countries per their reference.

7. the JIB approach and grade tables were great efforts, and referring to them is helpful but should have better suited in the supplementary material, as they are not the focus of this study, and rather bring more info from the supplementary to the main other the mere causes and mortality rate. Still can be included in a paragraph and two as a summary.

**Reviewer #2: ** This manuscript presents a systematic review and meta-analysis on traumatic brain injury (TBI) in Ethiopia. The study addresses a significant public health issue, given the high prevalence and associated challenges of TBI globally, especially in regions with limited healthcare resources like Ethiopia.

Strengths

Rigorous Research Methodology

The study strictly adheres to the Preferred Reporting Items for Systematic Reviews and Meta-Analyses (PRISMA) guidelines. From the comprehensive literature search across multiple databases (PubMed, Scopus, Web of Science, and EMBASE) using well-defined search terms and strategies, to the meticulous data extraction and quality assessment process, every step is methodologically sound. The employment of the Joanna Briggs Institute (JBI) critical appraisal checklist and the Grading of Recommendations Assessment, Development and Evaluation (GRADE) approach for evaluating study quality and evidence certainty further enhances the scientific rigor. The use of Stata 17 software and random-effects models to handle heterogeneity and assess publication bias is also appropriate and adds to the reliability of the findings.

Comprehensive Data Collection

The authors' efforts in conducting a thorough literature search, including both electronic database searches and manual reviews of reference lists, ensure a wide coverage of relevant studies. This comprehensive approach maximizes the likelihood of capturing all available evidence related to TBI in Ethiopia, strengthening the foundation of the analysis.

Weaknesses and Suggestions

Sample Representativeness Issue

The studies included in the review are disproportionately concentrated in certain regions, such as Addis Ababa. This uneven distribution may lead to a biased estimation of the national TBI burden, as rural and underserved areas might have different TBI patterns and risk factors. To address this, future research should prioritize large-scale, nationally representative studies. This could involve stratified sampling techniques to ensure adequate representation from all regions, including those that are currently under-researched. Additionally, collaborating with local healthcare providers and community organizations in different areas can facilitate data collection and improve the comprehensiveness of the sample.

Publication Bias Concern

By relying solely on published studies, the meta-analysis is susceptible to publication bias. Studies with significant results are more likely to be published, potentially skewing the overall picture. To mitigate this, the authors could consider exploring unpublished studies, such as those presented in conference proceedings or registered in trial databases. Grey literature searches can also be incorporated to capture a more complete range of research findings. This would provide a more balanced and accurate assessment of the TBI situation in Ethiopia.

Diagnostic Limitations

Many of the included studies lack access to advanced imaging modalities like CT or MRI, particularly in rural settings. This deficiency may result in the underreporting of TBI severity and complexity, as milder cases or subtle brain injuries could go undetected. Future investigations should aim to improve diagnostic capabilities in these areas. This could involve providing training and resources for healthcare workers to better identify and document TBI cases. Mobile diagnostic units equipped with appropriate imaging technology could also be deployed to reach rural and remote areas, ensuring more accurate diagnosis and a more comprehensive understanding of the true prevalence and nature of TBI.

Variability in Trauma Care Consideration

The variability in trauma care availability across Ethiopia is not adequately accounted for in the current analysis. Different levels of healthcare infrastructure and resources can significantly impact patient outcomes, including mortality and complication rates. To rectify this, future studies should incorporate trauma care availability as a variable in the analysis. This could involve collecting data on factors such as the number of trained healthcare providers, availability of critical care facilities, and access to emergency medical services in different regions. By adjusting for these factors, a more accurate assessment of the impact of TBI and the effectiveness of interventions can be achieved.

**Reviewer #3:**  The manuscript by Yohannis Derbew Molla and Hirut Tesfahun Alemu provides an essential and timely systematic review and meta-analysis on the prevalence, causes, and outcomes of TBI in Ethiopia. The study addresses a significant public health concern and highlights critical gaps in trauma care and prevention in a low-resource setting. The manuscript is well-organized and adheres to the PRISMA guidelines. However, several issues need to be addressed to enhance the clarity, accuracy, and impact of the study.

Major Comments

1. Authors acknowledge the data heterogeneity (e.g., I² values), they should explore subgroup analyses based on geographical regions, study design, or study population demographics to provide insights into the variability.

2. Include a funnel plot or Egger’s test results in the manuscript to substantiate the claim of publication bias assessment.

3. Clarify how mortality rates and complications were standardized across studies. Were these definitions uniform across studies, or did variability exist?

4. The lack of representation from certain Ethiopian regions, particularly rural and underserved areas, is a limitation. Highlight how this could impact the generalizability of findings.

5. Expand on the implications for Ethiopian health policy and trauma care systems. For example, how might these findings inform resource allocation or prevention strategies for road traffic accidents and violence?

Minor Comments

1. Rephrase ambiguous sentences for clarity, e.g., “... insufficient healthcare infrastructure... exacerbated by limited access to specialized medical services” could be refined for readability.

2. Include exact sample sizes (n) for all pooled estimates in tables and figures legends.

3. Verify the inclusion of all mandatory PRISMA elements, particularly in the supplementary material.

4. Provide additional details on statistical corrections for heterogeneity, if any were used.

5. Ensure recent references are cited for comparisons with other LMICs or African nations.

**********

6. PLOS authors have the option to publish the peer review history of their article (what does this mean? ). If published, this will include your full peer review and any attached files.

If you choose “no”, your identity will remain anonymous but your review may still be made public.

**Do you want your identity to be public for this peer review?** For information about this choice, including consent withdrawal, please see our Privacy Policy .

Reviewer #1: **Yes: ** Adel Elnashar

Reviewer #2: No

Reviewer #3: **Yes: ** Syed M. Faisal, Ph.D.

**********

[NOTE: If reviewer comments were submitted as an attachment file, they will be attached to this email and accessible via the submission site. Please log into your account, locate the manuscript record, and check for the action link "View Attachments". If this link does not appear, there are no attachment files.]

While revising your submission, please upload your figure files to the Preflight Analysis and Conversion Engine (PACE) digital diagnostic tool, https://pacev2.apexcovantage.com/ . PACE helps ensure that figures meet PLOS requirements. To use PACE, you must first register as a user. Registration is free. Then, login and navigate to the UPLOAD tab, where you will find detailed instructions on how to use the tool. If you encounter any issues or have any questions when using PACE, please email PLOS at figures@plos.org . Please note that Supporting Information files do not need this step.

# Author comment: Author response to Decision Letter 1

**DOI:** 10.1371/journal.pone.0322641.r003

29 Jan 2025

Response to Reviewers and Editor

We sincerely thank the reviewers and the editor for their thoughtful and constructive feedback on our manuscript, titled “Traumatic Brain Injury in Ethiopia: A Systematic Review and Meta-Analysis.” We greatly appreciate the time and effort invested in providing detailed comments, which have been invaluable in improving the quality and clarity of our work.

In this revised submission, we have carefully addressed all the comments raised by the reviewers and the editor in chief. Below, we provide a point-by-point response to each comment, outlining the revisions made and the rationale for these changes. We are confident that these revisions have strengthened the manuscript and aligned it more closely with the journal’s standards.

We look forward to your feedback and remain open to any further suggestions.

To Academic editor Dr. Ukachukwu

Comment 1:

"When submitting your revision, we need you to address these additional requirements. Please ensure that your manuscript meets PLOS ONE's style requirements, including those for file naming. The PLOS ONE style templates can be found at [link]."

Response:

Thank you for your guidance. We have tried to review the PLOS ONE style templates and ensured that our manuscript adheres to the journal's formatting requirements. We have tried to ensure that all elements of the manuscript align with the journal’s style and are ready for review.

Comment 2:

"Thank you for stating the following in your Competing Interests section: 'no'

Please complete your Competing Interests on the online submission form to state any Competing Interests. If you have no competing interests, please state 'The authors have declared that no competing interests exist.', as detailed online in our guide for authors."

Response:

Thank you for pointing this out. We confirm that the authors have no competing interests related to this study. Accordingly, we have updated the Competing Interests section in the manuscript to state:

"The authors have declared that no competing interests exist."

We have also included this statement in the cover letter for clarity. Please proceed with updating the online submission form on our behalf as indicated.

Comment 3:

"Please provide a complete Data Availability Statement in the submission form, ensuring you include all necessary access information or a reason for why you are unable to make your data freely accessible. If your research concerns only data provided within your submission, please write 'All data are in the manuscript and/or supporting information files' as your Data Availability Statement."

Response:

Thank you for your guidance. We have updated the Data Availability Statement to ensure compliance with PLOS ONE’s requirements. The statement now reads:

"All data are included in the manuscript and/or supporting information files."

If any additional clarifications are required regarding the Data Availability Statement, we are happy to provide further details.

Comment 4:

"Please include captions for your Supporting Information files at the end of your manuscript, and update any in-text citations to match accordingly. Please see our Supporting Information guidelines for more information."

Response:

Thank you for pointing this out. We have added captions for all Supporting Information files at the end of the manuscript, providing clear and concise descriptions for each file. Additionally, we have reviewed and updated all in-text citations to ensure they align with the Supporting Information guidelines provided by PLOS ONE. If any further adjustments or clarifications are required, we will be happy to make them.

Comment 5:

As required by our policy on Data Availability, please ensure your manuscript or supplementary information includes the following:

A numbered table of all studies identified in the literature search, including those that were excluded from the analyses.

For every excluded study, the table should list the reason(s) for exclusion.

Response:

Thank you for your insightful suggestion. We have added a detailed table summarizing the studies reviewed at the full-text screening stage, including the reasons for exclusion for studies not included in the final analysis. This table is now included in the supplementary file as Table 1.

Given the large number of studies initially identified (807 total), providing a table for every study, including those excluded at the title/abstract screening stage, would be impractical. Instead, we focused on the 41 studies assessed at the full-text review stage, as indicated in the PRISMA flowchart, and provided detailed reasons for excluding the 23 studies that did not meet our inclusion criteria.

We hope this approach aligns with your expectations, and we are happy to provide further details if needed.

Comment 6:

"If any of the included studies are unpublished, include a link (URL) to the primary source or detailed information about how the content can be accessed."

Response:

Thank you for your suggestion. None of the included studies in this systematic review and meta-analysis are unpublished. All studies were sourced from peer-reviewed journals and reputable databases, including PubMed, Scopus, Web of Science, and EMBASE. As such, no additional links or access information are required for unpublished studies.

Comment 7:

*A table of all data extracted from the primary research sources for the systematic review and/or meta-analysis. The table must include the following information for each study:

• Name of data extractors and date of data extraction

• Confirmation that the study was eligible to be included in the review

• All data extracted from each study for the reported systematic review and/or meta-analysis that would be needed to replicate your analyses.*

Response:

We thank the reviewer for highlighting this important point. In response, we have included a detailed table in our supplementary materials (see Table 2) that contains all the required information. Specifically:

1. Name of data extractors and date of data extraction:

o This information is clearly documented in the columns titled "Data Extractors" and "Date of data extraction."

2. Confirmation of eligibility for inclusion:

o The column "Article included in the study" explicitly indicates whether each study met the eligibility criteria for inclusion in the review.

3. All extracted data needed to replicate analyses:

o The table comprehensively details all relevant data extracted from each study, including study characteristics (e.g., design, region, demographics), outcome variables (e.g., mortality rate, complications, length of hospital stay), and other relevant metrics used in the meta-analysis.

We tried to ensure transparency by providing all necessary information to replicate the analyses presented in the manuscript. We trust this addresses your concerns.

Comment 8:

If data or supporting information were obtained from another source (e.g., correspondence with the author of the original research article), please provide the source of data and dates on which the data/information were obtained by your research group.

Response:

We appreciate the reviewer’s request for clarity on the sources of our data. All data included in this systematic review and meta-analysis were extracted directly from the published articles identified through our systematic search process. No additional data or supporting information were obtained through correspondence with authors or other external sources.

Comment 9:

If applicable for your analysis, a table showing the completed risk of bias and quality/certainty assessments for each study or outcome. Please ensure this is provided for each domain or parameter assessed. For example, if you used the Cochrane risk-of-bias tool for randomized trials, provide answers to each of the signaling questions for each study. If you used GRADE to assess certainty of evidence, provide judgments about each of the quality-of-evidence factors. This should be provided for each outcome.

Response:

Thank you for the insightful comment. We have provided detailed tables outlining the risk of bias and quality/certainty assessments for the included studies.

1. Risk of Bias Assessment:

o The risk of bias for each included study was evaluated using the Joanna Briggs Institute (JBI) critical appraisal checklist for observational studies.

o Table 1 in the manuscript outlines the responses to each of the eight questions from the JBI checklist for all included studies.

o The table provides a comprehensive assessment of risk across domains such as inclusion criteria, study population, measurement validity, and statistical methods.

2. Certainty of Evidence Assessment:

o The certainty of evidence was assessed using the GRADE approach.

o Table 3 in the manuscript summarizes the judgments for each GRADE domain (risk of bias, inconsistency, indirectness, imprecision, and publication bias) for the primary and secondary outcomes (e.g., prevalence, causes, complications, mortality).

These tables address all domains and parameters assessed, ensuring transparency in the evaluation of the included studies and outcomes.

Should further clarification or additional tables be required, we are happy to provide supplementary materials.

Comment 10:

An explanation of how missing data were handled. This information can be included in the main text, supplementary information, or relevant data repository. Please note that providing these underlying data is a requirement for publication in this journal, and if these data are not provided your manuscript might be rejected.

Response:

We appreciate the reviewer’s emphasis on the importance of transparency in handling missing data. In our systematic review and meta-analysis:

1. Identification of Missing Data:

o During the data extraction process, some studies did not report specific variables (e.g., demographic details, certain outcomes such as complications or imaging findings). These instances were documented and flagged during data collection.

2. Approach to Handling Missing Data:

o For studies where data on key outcomes were missing, these studies were excluded from the respective meta-analyses but were still considered in qualitative synthesis if they met the inclusion criteria.

o Missing data were not imputed to ensure the validity of pooled estimates and to avoid introducing bias.

3. Documentation of Missing Data:

o Tables summarizing the characteristics and results of included studies (e.g., Table 2 on the supplementary file ) explicitly indicate where data were not reported (e.g., marked as "NR" for "Not Reported").

o A detailed description of how missing data influenced the analysis is provided in the "Limitations" section of the Discussion.

4. Data Availability:

o All underlying data and extracted variables are provided in the supplementary materials to ensure full transparency and compliance with journal requirements.

If additional details or clarifications are needed, we will gladly include further explanations in the supplementary information or provide raw data via a relevant repository.

Comment 11:

PLOS authors have the option to publish the peer review history of their article (what does this mean?). If published, this will include your full peer review and any attached files. If you choose “no,” your identity will remain anonymous but your review may still be made public.

Response:

Thank you for providing this option. We choose to publish the peer review history of our article. We believe that sharing the peer review process will enhance the transparency and credibility of our work and contribute to the open dissemination of scientific knowledge.

Response to Dr. Adel Elnashar

Comment 1:

Please include more info regarding your strategy to collect the demographics and do the meta-analysis specifically in Ethiopia, which will be useful to anyone who is s a foreigner to the policies and census in Ethiopia. There was no details regarding how to you were able confirm these numbers. Resources and reference for confirmation would have been very helpful.

Response to Reviewer #1:

Thank you for your valuable feedback. We appreciate your suggestion to include more information regarding the strategy for collecting demographic data and conducting the meta-analysis, particularly for readers unfamiliar with Ethiopian policies and census data. Below, we have incorporated additional details into the methodology section of the manuscript. We have also provided references and clarified how the numbers were verified to enhance transparency and reproducibility.

Comment 2:

I understand you included references "at times", but why do you pick these studies, would have been a very helpful input

Response:

Thank you for your feedback. In addition to the database search, we also conducted a manual review of the reference lists of pertinent articles and reviews. This approach ensured the inclusion of studies that might not have been indexed in the primary databases. We have now elaborated on this process in the methodology section to provide more clarity.

Comment 3:

"Rewrite the demographics section. For example, stating that males are 78% and females are 22% is redundant. A more useful piece of info is how these populations in the different studies differed in every study when compared to the whole and why."

Response:

Thank you for your valuable feedback. We have rewritten the demographics section to provide a more meaningful analysis of the data. The revised section now highlights the gender and residence distribution across the included studies, emphasizing patterns and differences in rural versus urban settings and their association with TBI causes. This approach removes redundancy and adds interpretive context, aligning the demographic findings with the overarching themes of the manuscript. We hope the revised section addresses your concerns effectively. Please let us know if additional adjustments are required.

Comment 4:

"Please summarize the confidence intervals. I prefer this info to be in a table, for comparison. At times, it was wordy and not helpful."

Response:

Thank you for your feedback. We have addressed your suggestion by summarizing the key findings and their confidence intervals in a concise table format. This table facilitates direct comparison and eliminates redundancy, enhancing clarity and readability. The updated table has been incorporated into the results section (Table 2).

Comment 5:

Better describe figures. e.g. Figure 2: Forest plot of assault/violence as a cause of TBI in Ethiopia this should have been Figure 2: assault/violence as a cause of TBI in Ethiopia. Above is a Forest plot describing so and so.

Response:

Thank you for your insightful comment regarding the figure descriptions. We have revised all figure captions to provide more detailed explanations of their content and significance. These revised captions now describe the context, the type of data presented (e.g., pooled estimates and confidence intervals), and any notable observations, such as heterogeneity among studies. We believe these changes enhance the clarity and accessibility of the figures for the readers.

Comment 6:

I would suggest changing the name of the study e.g. "the prevalence of TBI in Ethiopia" or " comparing TBI prevalence in Ethiopia to the rest of sub-Saharan Africa", "The mechanism of TBI in Ethiopia" or whatever the authors prefer.

Response:

Thank you for your thoughtful suggestion regarding the title of the study. We agree that the title can be improved to better reflect the scope and focus of our review. In response, we have revised the title to "Epidemiology of Traumatic Brain Injury in Ethiopia: A Systematic Review and Meta-Analysis of Prevalence, Mechanisms, and Outcomes." This updated title highlights the key aspects of the study, ensuring clarity and alignment with the objectives of the review. We hope this change meets your expectations.

Comment 7:

There was no in depth analysis for the data in the supplementary material when it relates in Ethiopia, but only a referral to why the numbers are low in other countries per their reference.

Response:

Thank you for your valuable feedback. We appreciate your observation regarding the analysis of the

Attachment Submitted filename: Detailed response to the reviewers.docx

# Peer review: Decision Letter 1

**DOI:** 10.1371/journal.pone.0322641.r004

**Authors:** Alvan-Emeka K. Ukachukwu

7 Mar 2025

PONE-D-24-50934R1Epidemiology of Traumatic Brain Injury in Ethiopia: A Systematic Review and Meta-Analysis of Prevalence, Mechanisms, and OutcomesPLOS ONE

Dear Dr. Molla,

Thank you for submitting your manuscript to PLOS ONE. After careful consideration, we feel that it has merit but does not fully meet PLOS ONE’s publication criteria as it currently stands. Therefore, we invite you to submit a revised version of the manuscript that addresses the points raised during the review process.

==============================

**ACADEMIC EDITOR: See reviewer comments below.** ==============================

Please submit your revised manuscript by Apr 21 2025 11:59PM. If you will need more time than this to complete your revisions, please reply to this message or contact the journal office at plosone@plos.org . When you're ready to submit your revision, log on to https://www.editorialmanager.com/pone/ and select the 'Submissions Needing Revision' folder to locate your manuscript file.

Please include the following items when submitting your revised manuscript:

- A rebuttal letter that responds to each point raised by the academic editor and reviewer(s). You should upload this letter as a separate file labeled 'Response to Reviewers'.
- A marked-up copy of your manuscript that highlights changes made to the original version. You should upload this as a separate file labeled 'Revised Manuscript with Track Changes'.
- An unmarked version of your revised paper without tracked changes. You should upload this as a separate file labeled 'Manuscript'.

If you would like to make changes to your financial disclosure, please include your updated statement in your cover letter. Guidelines for resubmitting your figure files are available below the reviewer comments at the end of this letter.

If applicable, we recommend that you deposit your laboratory protocols in protocols.io to enhance the reproducibility of your results. Protocols.io assigns your protocol its own identifier (DOI) so that it can be cited independently in the future. For instructions see: https://journals.plos.org/plosone/s/submission-guidelines#loc-laboratory-protocols . Additionally, PLOS ONE offers an option for publishing peer-reviewed Lab Protocol articles, which describe protocols hosted on protocols.io. Read more information on sharing protocols at https://plos.org/protocols?utm_medium=editorial-email&utm_source=authorletters&utm_campaign=protocols .

We look forward to receiving your revised manuscript.

Kind regards,

Alvan-Emeka K. Ukachukwu, MD, MSc.GH

Academic Editor

PLOS ONE

Journal Requirements:

Please review your reference list to ensure that it is complete and correct. If you have cited papers that have been retracted, please include the rationale for doing so in the manuscript text, or remove these references and replace them with relevant current references. Any changes to the reference list should be mentioned in the rebuttal letter that accompanies your revised manuscript. If you need to cite a retracted article, indicate the article’s retracted status in the References list and also include a citation and full reference for the retraction notice.

Additional Editor Comments:

Please address the outstanding comments by the reviewer.

[Note: HTML markup is below. Please do not edit.]

Reviewers' comments:

Reviewer's Responses to Questions

**Comments to the Author**

1. If the authors have adequately addressed your comments raised in a previous round of review and you feel that this manuscript is now acceptable for publication, you may indicate that here to bypass the “Comments to the Author” section, enter your conflict of interest statement in the “Confidential to Editor” section, and submit your "Accept" recommendation.

Reviewer #1: (No Response)

Reviewer #3: All comments have been addressed

**********

2. Is the manuscript technically sound, and do the data support the conclusions?

The manuscript must describe a technically sound piece of scientific research with data that supports the conclusions. Experiments must have been conducted rigorously, with appropriate controls, replication, and sample sizes. The conclusions must be drawn appropriately based on the data presented. 

Reviewer #1: No

Reviewer #3: Yes

**********

3. Has the statistical analysis been performed appropriately and rigorously? 

Reviewer #1: Yes

Reviewer #3: Yes

**********

4. Have the authors made all data underlying the findings in their manuscript fully available?

The PLOS Data policy requires authors to make all data underlying the findings described in their manuscript fully available without restriction, with rare exception (please refer to the Data Availability Statement in the manuscript PDF file). The data should be provided as part of the manuscript or its supporting information, or deposited to a public repository. For example, in addition to summary statistics, the data points behind means, medians and variance measures should be available. If there are restrictions on publicly sharing data—e.g. participant privacy or use of data from a third party—those must be specified.

Reviewer #1: Yes

Reviewer #3: Yes

**********

5. Is the manuscript presented in an intelligible fashion and written in standard English?

PLOS ONE does not copyedit accepted manuscripts, so the language in submitted articles must be clear, correct, and unambiguous. Any typographical or grammatical errors should be corrected at revision, so please note any specific errors here.

Reviewer #1: Yes

Reviewer #3: Yes

**********

6. Review Comments to the Author

Please use the space provided to explain your answers to the questions above. You may also include additional comments for the author, including concerns about dual publication, research ethics, or publication ethics. (Please upload your review as an attachment if it exceeds 20,000 characters)

Reviewer #1: Thank you for your hard work reviewing the comments and rewriting. The quality of the work has much improved.

-Remove all forest plots and in a simple table list all the heterogeneity results for the different variables in question. The plot shows the heterogeneity, but in themselves don’t add much information, can be included in supplementary.

-in introduction, there is a consistent jump back and forth to the prevalence of TBI and the impact of TBI.

- The variable inconsistency between retrospective asnd prospective studies doesn’t indicate that what you mentioned. These variables are indeed expected to produce this effect, but your mentioned conclusion can’t be deducted from this input, unless the prospective studies shared population, methodology..etc. is this the case? include then.

-Having your own mortality rate definition is confusing, provide a different term.

-Your study has significant inconsistency, I2, this should your number one limitation, and the first recommendations should address this subject. Your discussion should also include it in your first to second paragraph as it reflects on all your input and conclusions.

-The paragraph with the heterogeneity and the publication bias, include each separately, they are unrelated, only include the numbers under results, and include your suggestions and reflections under discussions.

-Egger test coding can also be included under supplementary.

-Surgical intervention doesn’t reflect severity, indeed many of the most severe TBI aren’t warranted for surgery.Surgery is a tool used when it is indicated.A slight midline shift may indicate a surgery need, but a more severe bleed, may not benefit from surgery.

-Don’t use GCS to classify TBI severity in your study unless isolated, GCS can vary depending on general state, or otherwise list GCS ranges without referring to TBI severity.

-There was multiple outcomes variable added in your new manuscript. There was multiple versions, but after adding this, there was no consistency in using it elements for analysis. Please review these outcomes or remove.

Reviewer #3: The authors have adequately addressed and incorporated the requested information in the revised version of the manuscript.

**********

7. PLOS authors have the option to publish the peer review history of their article (what does this mean? ). If published, this will include your full peer review and any attached files.

If you choose “no”, your identity will remain anonymous but your review may still be made public.

**Do you want your identity to be public for this peer review?** For information about this choice, including consent withdrawal, please see our Privacy Policy .

Reviewer #1: **Yes: ** Adel Elnashar

Reviewer #3: **Yes: ** Syed M. Faisal

**********

[NOTE: If reviewer comments were submitted as an attachment file, they will be attached to this email and accessible via the submission site. Please log into your account, locate the manuscript record, and check for the action link "View Attachments". If this link does not appear, there are no attachment files.]

While revising your submission, please upload your figure files to the Preflight Analysis and Conversion Engine (PACE) digital diagnostic tool, https://pacev2.apexcovantage.com/ . PACE helps ensure that figures meet PLOS requirements. To use PACE, you must first register as a user. Registration is free. Then, login and navigate to the UPLOAD tab, where you will find detailed instructions on how to use the tool. If you encounter any issues or have any questions when using PACE, please email PLOS at figures@plos.org . Please note that Supporting Information files do not need this step.

# Author comment: Author response to Decision Letter 2

**DOI:** 10.1371/journal.pone.0322641.r005

10 Mar 2025

We sincerely thank the reviewers and the editor for their thoughtful and constructive feedback on our manuscript, titled “Epidemiology of Traumatic Brain Injury in Ethiopia: A Systematic Review and Meta-Analysis of Prevalence, Mechanisms, and Outcomes.” We greatly appreciate the time and effort invested in providing detailed comments, which have been invaluable in improving the quality and clarity of our work.

In this revised submission, we have carefully addressed all the comments raised by the reviewer. Below, we provide a point-by-point response to each comment, outlining the revisions made and the rationale for these changes.

We look forward to your feedback and remain open to any further suggestions.

Response to Dr. Adel Elnashar

Comment 1:

"Remove all forest plots and in a simple table list all the heterogeneity results for the different variables in question. The plot shows the heterogeneity, but in themselves don’t add much information, can be included in supplementary."

Response:

In response to your comment, we have removed all forest plots from the main text and replaced them with a comprehensive summary table that lists the pooled estimates, confidence intervals, heterogeneity (I²), and p-values for all key variables analyzed in the study (Table 3). This table provides a clear and concise overview of the heterogeneity results, making the findings more accessible to readers.

Additionally, all forest plots have been moved to the Supplementary Materials section, where they are labeled and referenced appropriately (Supplementary Figures 1–31). This change streamlines the main manuscript while ensuring that the detailed graphical representations remain available for readers who wish to explore the data further.

Comment 2:

"In the introduction, there is a consistent jump back and forth to the prevalence of TBI and the impact of TBI."

Response:

In response to your comment, we have thoroughly revised the Introduction to ensure a logical and coherent flow, avoiding unnecessary jumps between the prevalence and impact of TBI.

Comment 3:

"The variable inconsistency between retrospective and prospective studies doesn’t indicate that what you mentioned. These variables are indeed expected to produce this effect, but your mentioned conclusion can’t be deducted from this input, unless the prospective studies shared population, methodology, etc. Is this the case? Include then."

Response:

In response to your comment, we have revised the manuscript to provide a more nuanced interpretation of the heterogeneity between retrospective and prospective studies. Specifically, we have clarified that the lack of heterogeneity in prospective studies may be influenced by factors such as smaller sample sizes, similarities in population characteristics, and standardized data collection protocols, rather than inherent consistency in study design. For example, the prospective studies included in our analysis were conducted in similar urban settings and utilized comparable methodologies, which may have contributed to the observed consistency. We have also acknowledged that further research is needed to confirm these findings and explore additional factors that may influence heterogeneity. (see pages 7 (lines 240-247) and ).

Comment 4:

"Having your own mortality rate definition is confusing, provide a different term."

Response:

In response to your comment, we have revised the manuscript to use a more standard term for mortality rate. Specifically, we have replaced the custom definition with "short-term mortality rate", which we define as the proportion of patients who died due to traumatic brain injury (TBI) within the timeframe reported by each individual study (e.g., in-hospital mortality or 30-day mortality). This change improves clarity and ensures consistency with standard terminology in the field. The revised definition has been incorporated into the Methods (page 5, lines 163-166). Thank you for this insightful suggestion, which has enhanced the readability of the manuscript.

Comment 5:

"Your study has significant inconsistency, I2, this should your number one limitation, and the first recommendations should address this subject. Your discussion should also include it in your first to second paragraph as it reflects on all your input and conclusions."

Response:

In response to your comment, we have revised the manuscript to highlight heterogeneity (I²) as the primary limitation and address it early in the Discussion and Recommendations sections. Specifically, we have:

1. Moved the discussion of heterogeneity to the top of the Limitations section, emphasizing its impact on the generalizability and reliability of the findings (Page 17, lines 519-525).

2. Added a detailed discussion of heterogeneity in the first paragraph of the Discussion, including potential sources (e.g., regional variations, study design differences) and their implications for the interpretation of the results (Page 13, lines 353-358).

3. Made the first recommendation about addressing heterogeneity in future studies, suggesting strategies such as standardized data collection methods and stratified analyses (Page 17, lines 535-538).

Comment 6:

"The paragraph with the heterogeneity and the publication bias, include each separately, they are unrelated, only include the numbers under results, and include your suggestions and reflections under discussions."

Response:

We sincerely thank the reviewer for this valuable suggestion. In response to your comment, we have separated the discussion of heterogeneity and publication bias into distinct sections. Specifically, we have:

1. Included only the numerical results (e.g., I² values, Egger’s test p-values) in the Results section, presenting them in a clear and concise manner.

2. Moved the interpretation and reflections on heterogeneity and publication bias to the Discussion section, where we discuss their implications for the findings and recommendations for future research. (page 8/9, lines 303-313, Page 13, lines 353-363)

Comment 7:

"Egger test coding can also be included under supplementary."

Response:

We sincerely thank the reviewer for this suggestion. In response to your comment, we have moved the Egger’s test figures (e.g., funnel plots) to the Supplementary Materials

Comment 8:

"Surgical intervention doesn’t reflect severity, indeed many of the most severe TBI aren’t warranted for surgery. Surgery is a tool used when it is indicated. A slight midline shift may indicate a surgery need, but a more severe bleed, may not benefit from surgery."

Response:

We sincerely thank the reviewer for this important observation. In response to your comment, we have revised the manuscript to clarify that surgical intervention rates are not a direct measure of TBI severity but instead reflect the proportion of cases where surgery was clinically indicated (Page 7, 230-235, and Page 15, lines 467-475). Specifically, we have:

1. Emphasized that surgical intervention is based on specific clinical indications (e.g., epidural or subdural hematomas, midline shift, elevated intracranial pressure) rather than injury severity alone.

2. Discussed the variability in surgical intervention rates across studies, which may be influenced by differences in clinical practices, resource availability, and patient populations.

3. Provided context for the common indications for surgery in TBI and how these relate to the findings of our study.

Comment 9:

"Don’t use GCS to classify TBI severity in your study unless isolated, GCS can vary depending on general state, or otherwise list GCS ranges without referring to TBI severity."

Author Response:

In response to your comment, we have revised the manuscript to avoid using the Glasgow Coma Scale (GCS) to classify TBI severity

Comment 10:

There were multiple outcome variables added in your new manuscript. There were multiple versions, but after adding this, there was no consistency in using its elements for analysis. Please review these outcomes or remove."

Response:

We sincerely appreciate the reviewer’s careful assessment of our manuscript and their constructive feedback. We acknowledge the concern regarding multiple outcome variables and the need for consistency in their application throughout the analysis (page 5-8).

To address this, we have made the following modifications:

1. Clearly Defined Outcome Measures:

• We have categorized primary and secondary outcomes to ensure clarity and coherence.

• The primary outcomes include the pooled prevalence of TBI, leading causes (assaults, RTIs, falls), and overall mortality rate.

• The secondary outcomes now explicitly include GCS classification, complications (seizures, infections, aspiration pneumonia, wound infections), surgical intervention rate, imaging findings, and regional/demographic variations in TBI prevalence and outcomes.

2. Standardized Definitions for Key Outcomes:

• Short-term mortality is now clearly defined as the proportion of TBI-related deaths reported within each study’s specific follow-up period (e.g., in-hospital mortality or 30-day mortality). This standardization ensures comparability.

• Complications are now explicitly categorized based on available data, preventing inconsistencies in reporting.

3. Consistency in Subgroup Analyses:

• The subgroup analyses are now fully aligned with the defined outcome measures, ensuring coherence across different analyses.

• The surgical intervention rate is analyzed separately from TBI severity, clarifying that it reflects cases requiring neurosurgical procedures rather than serving as a direct severity indicator.

4. Addressing Heterogeneity and Variability:

• We have acknowledged and discussed the variability in study designs (retrospective, prospective, and cross-sectional), explaining how differences in methodology, population characteristics, and healthcare access contribute to heterogeneity.

• Regional differences are explored systematically, particularly in relation to mortality, complication rates, and surgical intervention rates, ensuring that the reported outcomes remain interpretable.

By implementing these revisions, we have improved the clarity, structure, and consistency of the outcome measures throughout the manuscript. We believe these changes effectively address the reviewer’s concern and enhance the rigor of our analysis.

We appreciate your valuable insights and welcome any further feedback to strengthen the manuscript.

Attachment Submitted filename: Detailed_response_to_the_reviewers_auresp_2.docx

# Peer review: Decision Letter 2

**DOI:** 10.1371/journal.pone.0322641.r006

**Authors:** Alvan-Emeka K. Ukachukwu

25 Mar 2025

Epidemiology of Traumatic Brain Injury in Ethiopia: A Systematic Review and Meta-Analysis of Prevalence, Mechanisms, and Outcomes

PONE-D-24-50934R2

Dear Dr. Yohannis Molla,

We’re pleased to inform you that your manuscript has been judged scientifically suitable for publication and will be formally accepted for publication once it meets all outstanding technical requirements.

Within one week, you’ll receive an e-mail detailing the required amendments. When these have been addressed, you’ll receive a formal acceptance letter and your manuscript will be scheduled for publication.

An invoice will be generated when your article is formally accepted. Please note, if your institution has a publishing partnership with PLOS and your article meets the relevant criteria, all or part of your publication costs will be covered. Please make sure your user information is up-to-date by logging into Editorial Manager at Editorial Manager®  and clicking the ‘Update My Information' link at the top of the page. If you have any questions relating to publication charges, please contact our Author Billing department directly at authorbilling@plos.org.

If your institution or institutions have a press office, please notify them about your upcoming paper to help maximize its impact. If they’ll be preparing press materials, please inform our press team as soon as possible -- no later than 48 hours after receiving the formal acceptance. Your manuscript will remain under strict press embargo until 2 pm Eastern Time on the date of publication. For more information, please contact onepress@plos.org.

Kind regards,

Alvan-Emeka K. Ukachukwu, MD, MSc.GH

Academic Editor

PLOS ONE

Additional Editor Comments (optional):

Reviewers' comments:

Reviewer's Responses to Questions

**Comments to the Author**

1. If the authors have adequately addressed your comments raised in a previous round of review and you feel that this manuscript is now acceptable for publication, you may indicate that here to bypass the “Comments to the Author” section, enter your conflict of interest statement in the “Confidential to Editor” section, and submit your "Accept" recommendation.

Reviewer #1: All comments have been addressed

**********

2. Is the manuscript technically sound, and do the data support the conclusions?

The manuscript must describe a technically sound piece of scientific research with data that supports the conclusions. Experiments must have been conducted rigorously, with appropriate controls, replication, and sample sizes. The conclusions must be drawn appropriately based on the data presented. 

Reviewer #1: Yes

**********

3. Has the statistical analysis been performed appropriately and rigorously? 

Reviewer #1: Yes

**********

4. Have the authors made all data underlying the findings in their manuscript fully available?

The PLOS Data policy requires authors to make all data underlying the findings described in their manuscript fully available without restriction, with rare exception (please refer to the Data Availability Statement in the manuscript PDF file). The data should be provided as part of the manuscript or its supporting information, or deposited to a public repository. For example, in addition to summary statistics, the data points behind means, medians and variance measures should be available. If there are restrictions on publicly sharing data—e.g. participant privacy or use of data from a third party—those must be specified.

Reviewer #1: Yes

**********

5. Is the manuscript presented in an intelligible fashion and written in standard English?

PLOS ONE does not copyedit accepted manuscripts, so the language in submitted articles must be clear, correct, and unambiguous. Any typographical or grammatical errors should be corrected at revision, so please note any specific errors here.

Reviewer #1: Yes

**********

6. Review Comments to the Author

Please use the space provided to explain your answers to the questions above. You may also include additional comments for the author, including concerns about dual publication, research ethics, or publication ethics. (Please upload your review as an attachment if it exceeds 20,000 characters)

Reviewer #1: Thank you for addressing my latest comments. Good luck!

I think a small map of Ethiopia would have been helpful to familiarize the reader with the geographical context of your work, especially if you are addressing an international audience. This won’t impact the core of the work or conclusion. Interested readers can seek a map on their own time. You have a couple of sentences that were redundant, but they are acceptable.

**********

7. PLOS authors have the option to publish the peer review history of their article (what does this mean? ). If published, this will include your full peer review and any attached files.

If you choose “no”, your identity will remain anonymous but your review may still be made public.

**Do you want your identity to be public for this peer review?** For information about this choice, including consent withdrawal, please see our Privacy Policy .

Reviewer #1: **Yes: ** Adel Elnashar

**********

# Editor report: Acceptance letter

**DOI:** 10.1371/journal.pone.0322641.r007

**Authors:** Alvan-Emeka K. Ukachukwu

PONE-D-24-50934R2

PLOS ONE

Dear Dr. Molla,

I'm pleased to inform you that your manuscript has been deemed suitable for publication in PLOS ONE. Congratulations! Your manuscript is now being handed over to our production team.

At this stage, our production department will prepare your paper for publication. This includes ensuring the following:

* All references, tables, and figures are properly cited

* All relevant supporting information is included in the manuscript submission,

* There are no issues that prevent the paper from being properly typeset

You will receive further instructions from the production team, including instructions on how to review your proof when it is ready. Please keep in mind that we are working through a large volume of accepted articles, so please give us a few days to review your paper and let you know the next and final steps.

Lastly, if your institution or institutions have a press office, please let them know about your upcoming paper now to help maximize its impact. If they'll be preparing press materials, please inform our press team within the next 48 hours. Your manuscript will remain under strict press embargo until 2 pm Eastern Time on the date of publication. For more information, please contact onepress@plos.org.

If we can help with anything else, please email us at customercare@plos.org.

Thank you for submitting your work to PLOS ONE and supporting open access.

Kind regards,

PLOS ONE Editorial Office Staff

on behalf of

Dr. Alvan-Emeka K. Ukachukwu

Academic Editor

PLOS ONE
